# Implications of adverse and benevolent childhood experiences on the physical and mental health of Mexican adults: a population-based study

**DOI:** 10.1016/j.lana.2025.101092

**Published:** 2025-04-19

**Authors:** Daniela León Rojas, Fabiola Castorena Torres, Nissa Yaing Torres-Soto, Irene Martín-Estal, Veronica Mundo Rosas, Brenda Martinez Tapia, Julieta Rodríguez-de-Ita

**Affiliations:** a Tecnologico de Monterrey, Escuela de Medicina y Ciencias de la Salud, Ave. Morones Prieto 3000, Monterrey, N.L, Mexico; b División de Ciencias de la Salud, Universidad Autónoma del Estado de Quintana Roo, Chetumal, Quintana Roo, Mexico; c Center for Evaluation and Survey Research, National Institute of Public Health, Mexico; d Tecnologico de Monterrey, Escuela de Medicina y Ciencias de la Salud, Hospital San José, TecSalud, N.L, Mexico

**Keywords:** Adverse childhood experiences, Benevolent childhood experiences, Global health, Mental health

## Abstract

**Background:**

Adverse childhood experiences (ACEs) are linked to negative physical and mental health outcomes. Limited information on their influence exists in Latin America and middle-income countries like Mexico. This study aimed to determine the prevalence and impact of ACEs and benevolent childhood experiences (BCEs) on Mexican population health.

**Methods:**

From September to November 2023, this cross-sectional study recruited a nationally representative sample of adults aged 18–65, randomly selected from urban and rural areas. Sociodemographic data, ACEs, BCEs, physical and mental health history, and clinical assessments for depression, anxiety, post-traumatic stress disorder, and eating disorders were collected.

**Findings:**

Of 1448 participants recruited, 1115 (77%) were women, 1278 (88·2%) reported at least one ACE, while 328 (22·6%) had four or more. Physical (840; 58·6%) and emotional neglect (518; 35·7%) were the most frequent. Four or more ACEs increased the odds of obesity (OR 1·8, 95% CI 1·2–2·8), hypertension (OR 1·6, 95% CI 1·1–2·2), depression (OR 4·7, 95% CI 3·6–6·1) and anxiety (OR 4·1, 95% CI 3·2–5·3) among others. Common BCEs included having at least one supportive caregiver (1298; 89·6%) and feeling comfortable with oneself (1272; 87·8%). BCEs decreased the odds of physical and mental health diagnoses.

**Interpretation:**

ACEs are highly prevalent and significantly impact the health of Mexican population. BCEs protect against these effects. Considering ACEs in public policies can help establish interventions to prevent adversity and promote positive childhood experiences.

**Funding:**

Fundación FEMSA, Centro de Primera Infancia from Tecnológico de Monterrey and Fundación FEMSA and Tecnologico de Monterrey Challenge-Based Research Funding Program 2022.

**Research in context**

**Evidence before this study**

Beginning in December 2022 and up to August 2023, we conducted a PubMed and Google Scholar search using the search terms “Adverse Childhood Experiences,” “Early Life Adversity,” “Benevolent Childhood Experiences,” “Positive Childhood Experiences,” “Mexico,” and “Latin America” with no limits on publication date or language. Several studies have focused on the effects of adverse childhood experiences (ACEs) on health, consistently demonstrating a linear dose–response relationship between the number of ACEs and health conditions, where having four or more ACEs increases the odds of physical and mental health outcomes. These associations persist even after controlling for demographic factors, with certain populations being more vulnerable due to social and economic conditions. On a counterpart, benevolent childhood experiences (BCE) have been associated with resilience and can counteract the negative effects of ACEs. Most ACE and BCE research has been conducted in North America and Europe, focusing less on Latin America and middle and low-income countries. There is significant variability in ACEs prevalence across different geographic, social, and cultural contexts, necessitating more research in underrepresented regions to understand their specific impacts on global health. Existing ACEs studies in Mexican adults lack nationwide representativeness, limiting their generalizability with even less information on BCEs.

**Added value of this study**

Studying ACEs and BCEs in a Latin American context, such as Mexico, can enhance understanding of childhood experiences and specific risks and protective factors within the socio-cultural context. This is the first study to report the prevalence of ACEs and BCEs in Mexican adults within a nationally representative sample, encompassing urban and rural areas and enabling cross-country comparisons within similar contexts. Focusing on a population often underrepresented in ACE studies, the findings contribute significantly to filling the knowledge gap regarding the Latin American population. Consequently, it aids in formulating screening methods, preventive strategies, and intervention proposals and developing informed national and international public policies to protect children from adversity.

**Implications of all the available evidence**

Adverse childhood experiences are highly prevalent in Mexico and exert a significant impact on health outcomes, while BCEs can mitigate their effects. Understanding the impact of childhood adversity in Mexico and other Latin American countries is crucial in the global effort to address and mitigate its effects. A comprehensive approach that considers ACEs and BCEs in child welfare policies, both nationally and internationally, can influence the formulation of interventions to prevent adversity, promote positive experiences for children in low and middle-income countries, and contribute to equity and inclusive child welfare.

## Introduction

Since Felitti's study in 1998, several studies have focused on the effects of adverse childhood experiences (ACEs)–such as abuse, neglect, and household dysfunction–on health. In the United States, the Behavioral Risk Factor Surveillance System has consistently demonstrated a linear dose–response relationship between the number of ACEs and health conditions, where having four or more ACEs increases the odds of physical and mental health outcomes.^1^ Even after controlling for demographic factors, higher odds of coronary heart disease, stroke, and diabetes have been linked to experiencing adversity. Additionally, adults with ACEs have shown higher relative risks for chronic obstructive pulmonary disease, cancer, depression, and substance abuse. Studies also highlight that while ACEs are across all populations, certain groups may be more vulnerable due to social and economic conditions.^2^

In contrast, benevolent childhood experiences (BCEs) represent protective influences that can counteract the maladaptive effects arising from ACEs.^3^ Although there is a recognized increase in the odds of poor health outcomes related to ACEs, many adults who have experienced adversity do not exhibit poor health or functioning. This underscores the non-deterministic nature of risk and the capacity of individuals and their social contexts to respond to adversity by mobilizing adaptive processes related to resilience.^4^ BCEs show a dose–response association in reducing the odds of having poor mental health in adults. They are also positively associated with young adult well-being, with their benefits persisting even when accounting for ACEs.^5^ This provides the rationale for conducting ACE studies that consider BCEs as moderating factors capable of attenuating or eliminating the association between ACEs and adverse outcomes.

A recent systematic review and meta-analysis by Madigan et al. highlights the predominance of ACE research in North America and Europe, with fewer studies conducted in Latin America and middle- and low-income countries. Significant variability has been observed in the prevalence of ACEs across different geographic, social, and cultural contexts, underscoring the need for more ACE studies in these countries to better understand their impact within the framework of global health research.^6^

Studying ACEs and BCEs within the Latin American social and cultural context, such as Mexico, can enrich the global understanding of how these factors shape childhood experiences, identify specific risks faced by the population, and reveal socio-cultural resources that can promote resilience. Mexico's social, cultural, and economic factors might influence exposure to adversity and positive childhood experiences. According to the 2022 National Institute of Statistics and Geography survey, 36·3% of the Mexican population lives in poverty, and the 2023 Global Organized Crime Index ranks Mexico third worldwide in criminality.^7^^,^^8^ Conversely, the family structure in the Mexican social context often provides support and safety, giving individuals a sense of belonging.^9^

Existing studies on ACEs among Mexican adults have reported their health impact, reporting higher odds of mental (depression, anxiety) and physical health (obesity, hypertension) diagnoses.^10^^,^^11^ However, these studies are limited by their focus on state-level data or specific populations, lack of information on BCEs, and the absence of nationwide representativeness, limiting their generalizability. Understanding the impact of adversity in Mexico and other Latin American countries is crucial for the global effort to address and mitigate the effects of ACEs. It can inform international policies and lead to the development of tailored strategies focused on screening, preventing adversity, promoting positive experiences in at-risk families, and reducing health costs in similar contexts globally. Mexico currently lacks population-level studies, clinical recommendations, and public health policies to address ACEs and even less information focused on BCEs. This study aims to conduct a national screening of ACEs and BCEs and determine their relationship with the physical and mental health status of the adult population in Mexico.

## Methods

### Study design

This cross-sectional study was conducted from September to November 2023, surveying a probabilistic, nationwide representative sample of Mexican adults aged 18 to 65 from urban and rural areas. A total of 1448 adults (690 urban and 758 rural) were analyzed, representing 75,461 adults nationwide (60,557 urban adults and 14,904 rural adults). The National Institute of Public Health ethics board approved the study protocol (CI:1860). All participants signed written informed consent forms. A mental health support line to provide psychological first aid, counseling, information, and referral to mental health services was available for all participants during the study. Information regarding ACEs, BCEs, and mental health services was provided to all participants.

### Participants

The inclusion criteria required participants to be between 18 and 65 years old. The exclusion criteria included intellectual disability that limited understanding of the data collection instruments and incomplete assessment.

### Procedures

Trained interviewers visited the randomly selected households, collected sociodemographic data, and administered internationally standardized questionnaires on ACEs and BCEs. They also gathered information on self-reported physical and mental health diagnoses and conducted clinical assessments for mental health.

### Sociodemographic data

Participants self-reported their marital status, educational level, and employment status. Data on belonging to specific ethnic groups was not collected due to language, scale translation, and validation barriers. Sex was self-declared and recorded as a binary variable with the options of male and female. Age was calculated in years as of the interview date. Localities with fewer than 2500 inhabitants were classified as rural areas, while those with 2500 or more were classified as urban.^12^

### Well-being index

The Well-Being Index (WBI) was estimated through principal component analysis based on housing characteristics and material possessions. It was categorized into tertiles, with one being the lowest or most economically needy stratum and three being the highest or least economically needy stratum.^13^

### Food security

Food security was determined using the Latin American and Caribbean Food Security Scale (ELCSA). This scale inquires whether the respondent or any household member has experienced a specific manifestation of food insecurity in the previous three months. Outcomes are classified as food security, mild food insecurity, moderate food insecurity, and severe food insecurity.^14^

### Adverse childhood experiences

ACEs were assessed using the ACE-IQ by the World Health Organization, which includes 13 categories of adversity such as physical abuse, emotional abuse, sexual abuse, household substance abuse, incarcerated household member, household member with chronic depression, mental illness, institutionalization, or suicidal behavior, household member treated violently, parental absence, separation or divorce, emotional neglect, physical neglect, bullying, community violence, and collective violence. Each adversity was recorded as present or absent, resulting in a total score ranging from 0 to 13, according to the ACE-IQ scoring manual.^15^ The Chilean Spanish version of ACE-IQ was revised and adapted for the Mexican context. Then, a confirmatory factor analysis was conducted, which resulted in high and significant factor loadings (p < 0·05). The model demonstrated a multifactorial structure supported by statistical goodness-of-fit indicators (χ2 = 528·562 [140 df], p < 0·001, relative χ2 = 3·77), practical (BBNFI = 0·98, BBNNFI = 0·95, CFI = 0·98), and population (RMSEA = 0·07).

### Benevolent childhood experiences

BCEs were assessed with the BCEs scale, a 10-item self-report instrument designed to assess childhood experiences. These include having stable eating or sleeping routines, caregivers who provide support and care, supportive teachers or other adults (not parents), enjoying school, opportunities for fun, good neighbors and friends, comforting beliefs, and being comfortable with oneself. The score ranges from 0 to 10, based on the sum of positive childhood experiences reported by the respondent. The BCE scale was developed in English and Spanish and was applied to Latin and Mexican participants in the United States.^3^ The original Spanish version was revised. Then, a confirmatory factor analysis was performed, which resulted in high and significant factor loadings (p < 0·05). The model demonstrated a unifactorial structure supported by statistical goodness-of-fit indicators (χ2 = 523·629 [141 df], p < 0·001, relative χ2 = 3·71), practical (BBNFI = 0·95, BBNNFI = 0·96, CFI = 0·97), and population (RMSEA = 0·06).

### Self-reported physical and mental health

Participants were asked to provide information about their physical and mental health status, including any physician-diagnosed conditions throughout their lifetime. Based on a literature review regarding prevalence and association with adversity, participants reported cardiovascular, metabolic, digestive, respiratory, rheumatologic, neurologic, oncologic, and psychiatric diagnoses. An additional section allowed for reporting other diagnoses.

### Clinically assessed mental health diagnoses

Depression: The PHQ-9 is a nine-item self-report questionnaire assessing the presence of depression symptoms. Each item is rated on a scale of 0–3, indicating the frequency of symptoms. The total score ranges from 0 to 27, with severity classified as follows: 5–9 (mild), 10–14 (moderate), 15–19 (moderately severe), and 20–27 (severe). Mexican Spanish version Cronbach alpha is 0·89.^16^

Generalized anxiety: The GAD-7 is a seven-item questionnaire that evaluates anxiety symptoms over the past two weeks. Responses are rated on a scale from 0 to 3, indicating symptom frequency, with a total score range of 0–21. Anxiety severity is classified based on total scores: 5–9 (mild), 10–14 (moderate), and >15 (severe). Mexican Spanish version Cronbach alpha is 0·93.^17^

Posttraumatic stress disorder: The PCL-5 is a 20-item questionnaire used to assess the presence and severity of PTSD symptoms according to DSM-5 criteria. Participants rate each symptom's severity over the past month on a scale from 0 (not at all) to 4 (extremely). The total score ranges from 0 to 80, with a provisional PTSD diagnosis made using a cut-off score of 33. Mexican Spanish version Cronbach alpha is 0·97.^18^

Eating disorders: The SCOFF Questionnaire is a five-item questionnaire designed to evaluate core features of anorexia and bulimia nervosa. Each question is answered dichotomously (yes or no), with scores ranging from 0 to 5 based on the sum of positive responses. A score of two or more indicates a suspicion of an eating disorder. Mexican Spanish version Cronbach alpha is 0·86.^19^

### Statistical analysis

The sample size was designed to represent Mexico's population aged 18–65, considering a 5% estimation error, 95% confidence, a 30% nonresponse rate, and a design effect of 2. The minimum estimated sample size was 1200 participants. For data analysis, sociodemographic data were described using frequencies and percentages for categorical variables. Regarding ACEs and BCEs prevalence, a comparative analysis between sex (women vs. male) and urban and rural strata data was conducted using contingency tables, the chi-squared test (X^2^), and Fisher's exact test. Logistic regression models were used to predict the presence of self-reported physical diagnoses and mental health diagnoses (self-reported and clinically assessed) based on the cumulative number of ACEs. Due to the small number of individuals reporting diagnoses such as multiple sclerosis, obstructive sleep apnea, fibromyalgia, hyperthyroidism, and mental health diagnoses like schizophrenia, bipolar disorder, substance abuse disorder, and eating disorders, these were excluded from the analysis. Raw odds ratios and 95% confidence intervals were reported. Statistical significance was determined with a p-value <0·05. Models were then adjusted for sociodemographic data, including age, sex, urbanity stratum, and wellness condition. An additional adjustment was performed using the BCEs score to determine the effect of benevolent childhood experiences. Adjusted odds ratios and 95% confidence intervals were reported. A Bonferroni correction was applied using all terms in each adjusted model. Statistical significance was determined with a p-value <0·01. The linearity of the continuous variables with respect to the logit of the dependent variable was assessed via the Box-Tidwell procedure. All statistical analyses were conducted using SPSS V.29.0.

### Role of the funding source

The study's funders had no role in the design of this study, analysis, data interpretation, or report writing.

## Results

A total of 1448 adults aged 18 to 65 were recruited for the study. Among the participants, 747/1448 (51·5%) were aged 18–39, 1115/1448 (77%) were women and 333/1448 (23%) were men. Of them, 658/1448 (45·4%) were married, and 1140/1390 (82·0%) had elementary, middle, or high-school degrees. Additionally, 697/1448 (48·1%) were employed. Participants were distributed across three wellness conditions strata, with 485/1448 (33·4%) in the most economically needy stratum and 613/1448 (42·3%) experiencing food security. Rural areas had a higher proportion of individuals without formal education and those in the most economically needy stratum. Conversely, urban areas had higher levels of food security and more individuals with higher educational degrees. Table 1 provides the demographic description of the population.

**Table 1:** Sociodemographic characteristics of Mexican adults aged 18 to 65 compared by urban and rural strata.^f^

	National population^a^		Urban population^b^		Rural population^c^		p^d^
	N = 1448	%	N = 690	%	N = 758	%	
**Population**							
Adults 18–65 years old	1448	100	690	80·2	758	17·8	
**Age**							0·001
18–39 years	747	51·5	324	46·9	423	55·8	
40–59 years	520	35·9	257	37·2	263	34·6	
60 years or more	181	12·5	109	15·7	72	9·4	
**Sex**							0·16
Masculine	333	23·0	169	24·5	164	21·6	
Feminine	1115	77·0	521	75·5	594	78·3	
**Marital status**							<0·001
Single	257	19·0	155	22·4	102	13·4	
Living with partner	365	25·2	147	21·3	218	28·7	
Married	658	45·4	305	44·2	353	46·5	
Separated	67	4·6	23	3·3	44	5·8	
Divorced	40	2·7	28	4·0	12	1·6	
Widowed	61	4·2	32	4·6	29	3·8	
**Laboral status**							<0·001
Employed	697	48·1	362	52·4	335	44·1	
Looking for a job	20	1·4	15	2·1	5	0·6	
Unemployed	58	4·0	36	5·2	22	3·0	
Housework	641	44·2	249	36·0	392	51·7	
Retired	32	2·2	28	4·0	4	0·5	
**Scholar degree**^e^							<0·001
No scholarship	137	9·9	41	6·1	96	13·3	
Basic	1140	82·0	540	80·9	600	82·9	
Professional or more	113	8·1	86	12·9	27	3·7	
**Wellness condition**							<0·001
Tercile 1 (low)	485	33·4	103	14·9	382	50·3	
Tercile 2 (medium)	480	33·1	239	34·6	241	31·7	
Tercile 3 (high)	483	33·3	348	50·4	135	17·8	
**Food security**							<0·001
Security	613	42·3	338	48·9	275	36·2	
Mild insecurity	516	35·6	238	34·4	278	36·6	
Moderate insecurity	178	12·3	62	8·9	116	15·3	
Severe insecurity	141	9·7	52	7·5	89	11·7	

Statistical significance was determined with a p-value <0·05.

a Total population = 1448 adults, representing 75,461 nationwide.

b Urban population = 690 adults representing 60,557 nationwide.

c Rural population = 758 adults representing 14,904 nationwide.

d X^2^.

e 58 out of 1448 participants with missing data.

f Localities with 2500 or more inhabitants were considered urban, and localities with <2500 were considered rural areas.

**Fig. 1:**
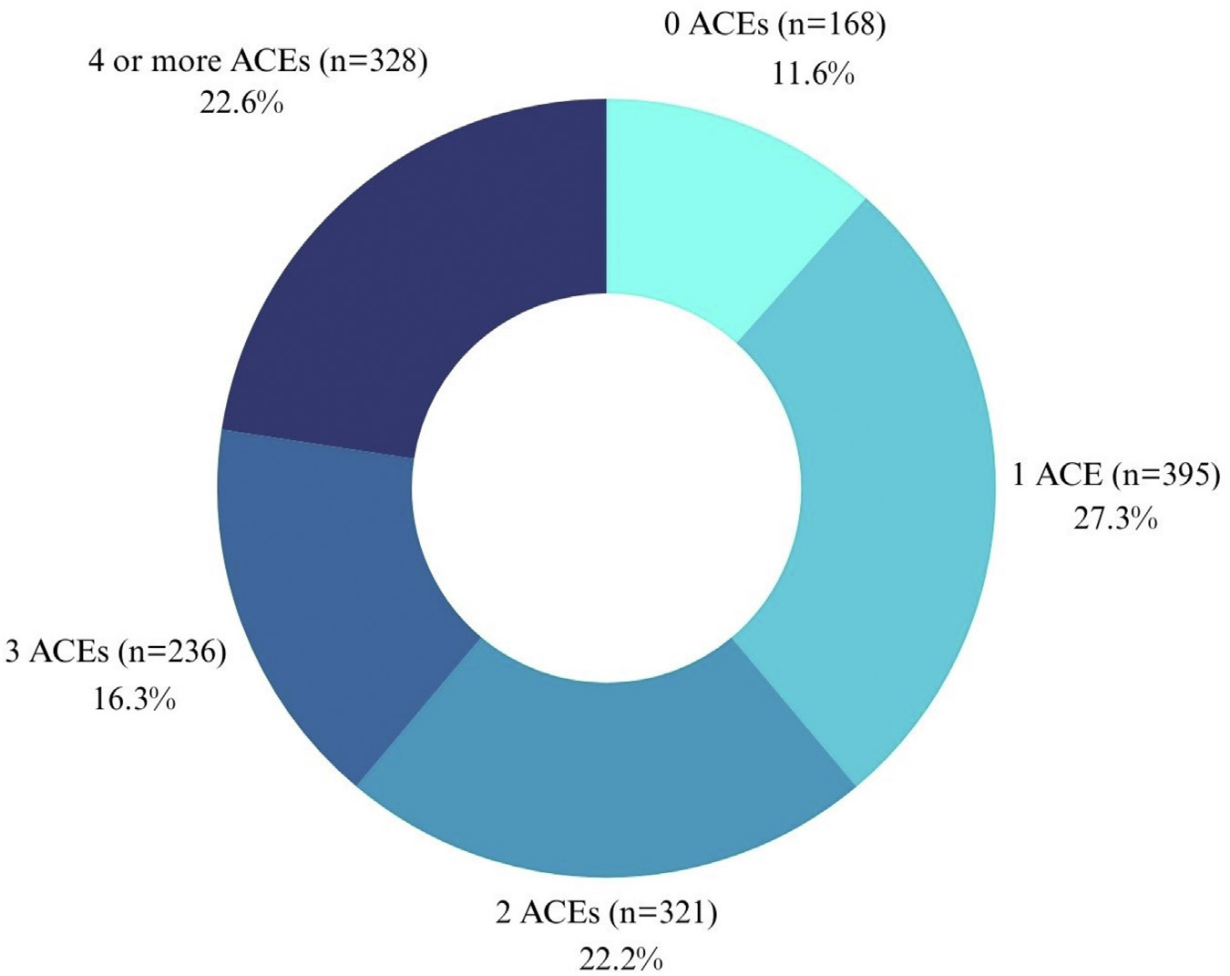
Prevalence of adverse childhood experiences (ACEs) in Mexican adults.

**Fig. 2:**
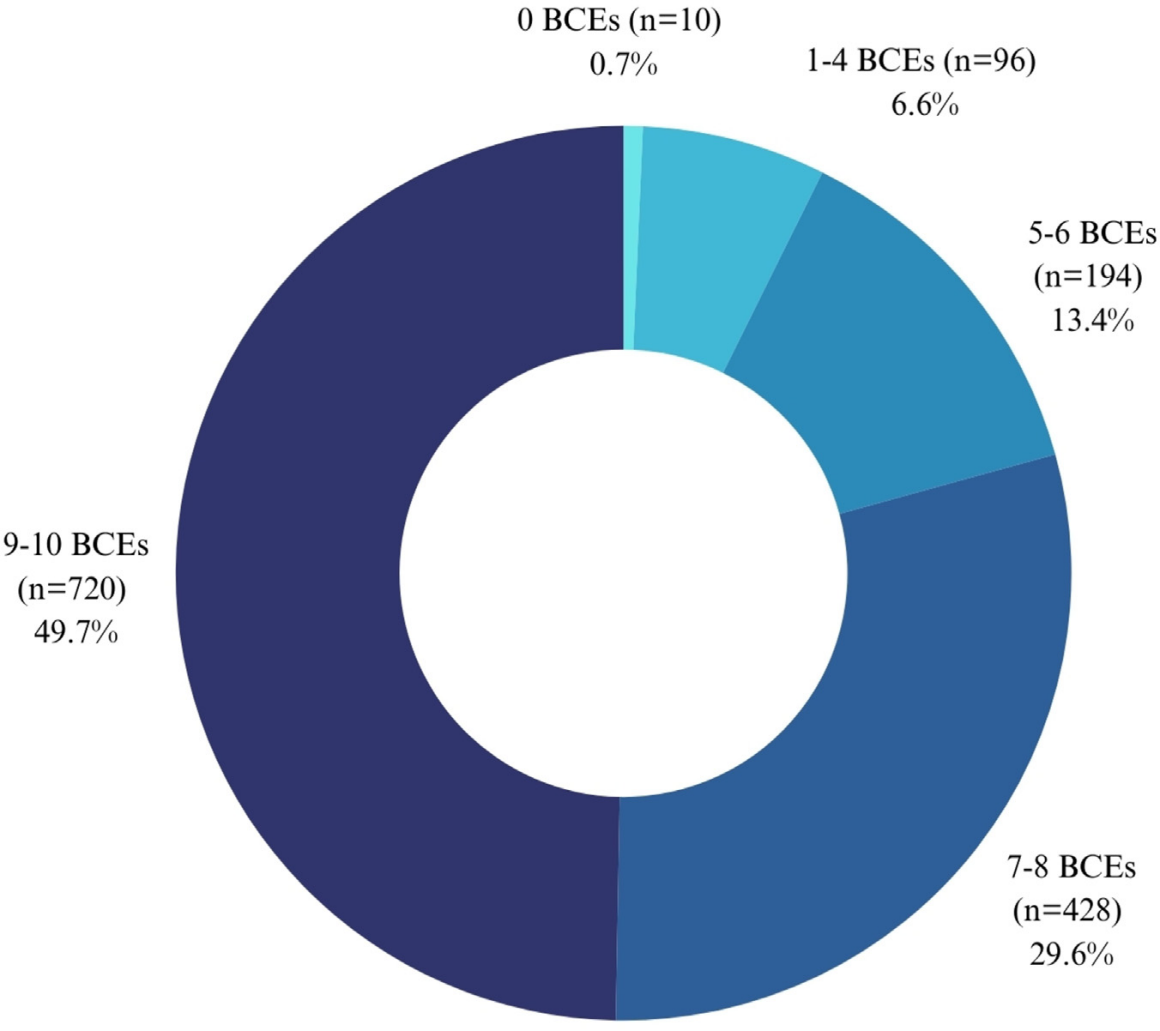
Prevalence of benevolent childhood experiences (BCEs) in Mexican adults.

In general, 1278/1448 (88·2%) participants (979/1278 women and 299/1278 men) had at least one ACE, while 328/1448 (22·6%), with 258/328 women and 68/328 men in this group, having four or more. Additionally, 720/1448 (49·7%) participants (545/720 women and 177/720 men) reported 9-10 BCEs, while only 10/1448 (0·7%), consisting of seven women and three men, reported zero BCEs. Comparison by sex revealed no significant differences in the reporting (Fig. 1, Fig. 2).

According to type, the most frequently reported ACEs were physical neglect (840/1448, 58·6%), emotional neglect (518/1448, 35·7%), and parental absence (480/1448, 33·1%), with parental absence being significantly more prevalent in urban areas. Collective violence was the least reported ACE (34/1448, 2·4%), and it was significantly more prevalent in urban areas (Table 2). Sexual abuse was significantly more reported by women, while collective and community violence were more reported by men. The most frequently reported BCEs were having at least one caregiver with whom they felt safe (1298/1448, 89·6%), liking or feeling comfortable with themselves (1272/1448, 87·8%) and enjoying school (1244/1448, 86%). ‘Enjoying school’ was reported more often by women. The opportunities to have a good time and having at least one good friend were significantly more reported by men. Likewise, these two and having a supportive teacher were significantly more reported in urban areas (Table 2). See Supplementary Table S1 for complete ACEs and BCEs type comparisons by sex.

**Table 2:** Prevalence of ACEs and BCEs in Mexican adults aged 18 to 65: urban vs. rural comparison.^e^

	National population^a^			Urban population^b^			Rural population^c^			p^d^
	N	%	95% CI	N	%	95% CI	N	%	95% CI	
**ACEs**										
Physical neglect	840	58·6	(54–63·1)	401	58·1	(53·2–64·2)	439	57·9	(53·2–62·8)	0·95
Emotional neglect	518	35·7	(31·8–39·7)	260	37·6	(31·7–41·2)	258	34·0	(29·0–37·7)	0·15
Parental separation or divorce	480	33·1	(30·3–38·7)	246	35·6	(30·1–40·3)	234	30·8	(27·4–36·3)	**0·04**
Household member treated violently	389	26·8	(21·4–27·9)	170	24·6	(19·6–27·5)	219	28·9	(25·5–33·3)	0·07
Alcohol or drug abuser in the household	374	25·8	(19·3–26·6)	163	23·6	(17·3–26·3)	211	27·8	(24·4–31·5)	0·07
Sexual abuse	211	14·5	(13·7–20·2)	106	15·3	(13·7–21·6)	105	13·9	(11·8–17·1)	0·39
Community violence	143	9·8	(7·7–11·2)	75	10·8	(7·6–11·8)	68	8·9	(6·4–11·1)	0·22
Emotional abuse	134	9·2	(6·9–10·8)	61	8·8	(6·5–11·1)	73	9·6	(7·1–12·4)	0·61
Mental disorders within the household	133	9·2	(7·6–11·3)	70	10·1	(7·5–12·1)	63	8·3	(6·1–10·9)	0·19
Bullying	110	7·6	(7·4–11·9)	62	9·0	(7·9–13·3)	48	6·3	(4·2–8·8)	0·06
Physical Abuse	105	7·2	(4·9–9·1)	46	6·6	(4·5–9·7)	59	7·7	(5·1–9·8)	0·37
Incarcerated household member	85	5·8	(4·3–8·3)	45	6·5	(4·1–9·0)	40	5·2	(3·9–8·1)	0·34
Collective violence	34	2·4	(2·0–4·7)	25	3·6	(2·2–5·6)	9	1·2	(0·6–2·7)	**0·002**
**BCEs**										
At least one caregiver whom you felt safe	1298	89·6	(88·1–92·6)	618	89·5	(87·7–93·1)	680	89·9	(86·5–92·5)	0·92
Liking or feeling comfortable with yourself	1272	87·8	(86·0–90·3)	606	87·8	(85·1–90·4)	666	87·9	(86·9–91·4)	0·94
Enjoying school	1244	86·0	(83·5–89·3)	601	87·1	(83·0–90·1)	643	84·8	(82·3–88·5)	0·15
Having good neighbors	1221	84·3	(81·0–86·6)	584	84·6	(79·9–86·7)	637	84·1	(82·8–88·5)	0·62
Having predictable home routines	1181	81·6	(77·7–85·1)	556	80·5	(76·1–85·2)	625	82·4	(80·6–86·8)	0·54
Having opportunities to have a good time	1122	77·4	(77·6–83·0)	559	81·0	(78·5–84·9)	563	74·3	(70·6–77·7)	**0·05**
Adult (not parent or caregiver) providing support	1108	76·5	(75·1–81·4)	540	78·2	(74·8–82·4)	568	75·0	(73·0–80·2)	0·12
At least one good friend	1083	74·8	(73·1–79·8)	536	77·6	(73·2–81·3)	547	72·1	(68·6–77·0)	**0·01**
Beliefs that gave you comfort	1061	73·3	(70·1–77·4)	506	73·3	(69·1–78·0)	555	73·2	(69·3–78·9)	0·85
At least one teacher who cared about you	899	62·0	(61·2–68·3)	454	65·8	(62·2–70·6)	445	58·7	(53·2–62·6)	**0·004**

Statistical significance was determined with a bolded p-value <0·05.

a Total population = 1448 adults, representing 75,461 nationwide.

b Urban population = 690 adults representing 60,557 nationwide.

c Rural population = 758 adults representing 14,904 nationwide.

d X^2^.

e Localities with 2500 or more inhabitants were considered urban, and localities with <2500 were considered rural areas.

Table 3 shows that the most prevalent physical diagnoses reported by participants were hypertension (203/1448, 14%), migraine (151/1448,10·4%), obesity (112/1448,7·7%), diabetes (135/1448,9·3%), and irritable bowel syndrome (111/1448, 7·6%). Hypertension, insomnia, asthma, and hypothyroidism were significantly more common in urban areas. Regarding mental health diagnoses and symptoms, 363/1448 (25%) participants reported having depression, 105/1448 (7·2%) reported anxiety, 64/1448 (4·4%) had suicidal ideation, 63/1448 (4·3%) reported alcoholism, and 52/1448 (3·6%) had PTSD. Clinical assessment revealed 490/1448 (33·8%) of participants with depression, 436/1448 (30·1%) with generalized anxiety disorder, 364/1448 (25·1%) with eating disorders, and 136/1448 (9·3%) with PTSD.

**Table 3:** Prevalence of Physical (self-reported) and mental health (self-reported and clinically assessed) diagnoses in Mexican adults aged 18 to 65: urban vs. rural comparison.^e^

	National population^a^			Urban population^b^			Rural population^c^			p^d^
	N	%	95% CI	N	%	95% CI	N	%	95% CI	
**Physical health self-report**										
Hypertension	203	14·0	(12·4–18·)	110	16·0	(12·5–20·2)	93	12·2	(9·5–16·9)	**0·03**
Diabetes mellitus	135	9·3	(7·1–11·5)	71	10·2	(6·9–12·3)	64	8·4	(6·5–11·3)	0·15
Obesity	112	7·7	(7·2–13·2)	61	8·8	(7·1–14·3)	51	6·7	(4·9–13·5)	0·09
Dyslipidemia	59	4·1	(3·3–7·1)	33	4·7	(3·2–7·9)	26	3·4	(2·4–6·6)	0·13
Cardiovascular disease	30	2·1	(1·6–4·5)	17	2·5	(1·6–5·2)	13	1·7	(1·0–3·4)	0·14
Irritable bowel syndrome	111	7·6	(6·7–11·5)	61	8·8	(6·6–12·5)	50	6·5	(5·4–10·4)	0·08
Gastroesophageal reflux disease	101	6·9	(5·6–9·5)	52	7·5	(5·5–10·2)	49	6·5	(4·8–8·9)	0·39
Migraine headache	151	10·4	(8·6–12·7)	73	10·5	(8·2–13·1)	78	10·3	(8·1–14·3)	0·75
Epilepsy	11	0·7	(0·3–1·4)	7	1·0	(0·4–1·7)	4	0·5	(0·1–1·1)	0·08
Multiple sclerosis	1	0·06	(0·0–0·2)	0	0·0	(0·0–0·0)	1	0·1	(0·0–0·9)	…
Insomnia	52	3·6	(3·1–6·7)	32	4·6	(3·3–7·7)	20	2·6	(1·6–4·0)	**0·03**
Obstructive sleep apnea	4	0·2	(0·0–0·2)	1	0·1	(0·0–0·2)	3	0·4	(0·1–1·0)	…
Chronic pain	80	5·5	(4·3–7·7)	33	4·7	(3·8–7·8)	47	6·2	(4·9–10·7)	0·39
Arthritis	22	1·5	(1·1–3·3)	13	1·8	(1·1–3·8)	9	1·1	(0·6–2·1)	0·17
Fibromyalgia	7	0·5	(0·2–1·1)	4	0·5	(0·2–1·3)	3	0·4	(0·1–2·0)	…
Chronic obstructive pulmonary disease	31	2·1	(1·2–3·3)	18	2·6	(1·2–3·8)	13	1·7	(0·8–2·7)	0·18
Asthma	30	2·1	(2·0–5·8)	21	3·0	(2·3–6·9)	9	1·1	(0·7–2·8)	**0·01**
Hypothyroidism	30	2·1	(1·5–3·8)	20	2·8	(1·6–4·5)	10	1·3	(0·5–2·5)	**0·03**
Hyperthyroidism	12	0·8	(0·7–2·9)	8	1·1	(0·7–3·5)	4	0·5	(0·2–1·7)	…
Cancer	17	1·1	(1–3·4)	12	1·7	(1–4)	5	0·6	(0·4–3·1)	0·06
**Mental health self-repot**										
Depression	363	25·0	(22·2–29·1)	174	25·2	(21·5–30·2)	189	24·9	(21·1–30·3)	0·62
Bipolar disorder	22	1·5	(0·7–2·5)	9	1·3	(0·5–2·7)	13	1·7	(1·0–4·1)	0·50
Suicidal ideation	64	4·4	(3·6–7·9)	35	5·0	(3·6–8·8)	29	3·8	(2·8–5·7)	0·29
Non-suicidal self harm	28	1·9	(1·6–4·5)	18	2·6	(1·8–5·4)	10	1·3	(0·5–2·2)	0·08
Generalized anxiety disorder	105	7·2	(7·0–11·5)	62	9·0	(7·5–13·2)	43	5·6	(3·3–7·3)	**0·02**
Posttraumatic stress disorder	52	3·6	(2·6–5·9)	28	4·0	(2·6–6·7)	24	3·1	(1·6–4·4)	0·38
Alcoholism	63	4·3	(3·1–5·9)	31	4·5	(2·9–6·3)	32	4·2	(2·8–6·3)	0·86
Any substance abuse disorder	13	0·9	(0·5–1·7)	9	1·3	(0·5–1·9)	4	0·5	(0·2–2·1)	0·12
Attention deficit and hyperactivity disorder	19	1·3	(0·8–3·2)	11	1·6	(0·8–3·8)	8	1·0	(0·4–2·4)	0·39
Anorexia nervosa	10	0·6	(0·3–1·3)	7	1·0	(0·3–1·6)	3	0·4	(0·1–1·2)	0·16
Bulimia nervosa	7	0·5	(0·1–1·0)	3	0·4	(0·1–1·3)	4	0·5	(0·2–1·4)	0·77
Schizophrenia	7	0·5	(0·2–1·3)	5	0·7	(0·2–1·6)	2	0·2	(0·0–0·7)	0·21
Borderline personality disorder	6	0·4	(0·1–0·5)	2	0·2	(0·0–0·6)	4	0·5	(0·1–1·1)	0·47
**Mental health clinically assessed**										
Depression (PHQ-9)	490	33·8	(29·9–35)	230	33·3	(29·5–36·4)	260	34·3	(28·9–36·5)	0·59
Generalized anxiety disorder (GAD-7)	436	30·1	(27·0–33)	198	28·6	(26·4–31·6)	238	31·3	(30·8–33·6)	0·19
Eating disorders (SCOFF)	364	25·1	(20·1–26)	165	23·9	(19·2–25·4)	199	26·2	(25·6–29·8)	0·24
Posttraumatic stress disorder (PCL-5)	136	9·3	(8·0–10·5)	64	9·2	(8·7–10·6)	72	9·5	(8·6–10·1)	0·83

Statistical significance was determined with a bolded p-value <0·05.

a Total population = 1448 adults, representing 75,461 nationwide.

b Urban population = 690 adults representing 60,557 nationwide.

c Rural population = 758 adults representing 14,904 nationwide.

d X^2^.

e Localities with 2500 or more inhabitants were considered urban, and localities with <2500 were considered rural areas.

Having four or more ACEs was associated with migraines (OR 1·7, 95% CI 1·2–2·5), diabetes (OR 1·5, 95% CI 1·0–2·2), obesity (OR 1·8, 95% CI 1·2–2·8), hypertension (OR 1·6, 95% CI 1·1–2·2), irritable bowel syndrome (OR 1·8, 95% CI 1·2–2·7), gastroesophageal reflux (OR 1·6, 95% CI 1·0–2·5), chronic pain (OR 2·0, 95% CI 1·2–3·2), and insomnia (OR 3·2, 95% CI 1·8–5·6). Additionally, there was an association between having four or more ACEs and self-reported diagnoses of depression (OR 5·3, 95% CI 3·2–8·5), PTSD (OR 3·1, 95% CI 1·7–5·4), anxiety (OR 3·3, 95% CI 1·5–6·9), suicidal ideation (OR 8·6, 95% CI 4·9–14·8), non-suicidal self harm (OR 5·5, 95% CI 2·5–11·9), alcoholism (OR 2·8, 95% CI 1·7–4·8), and attention deficit and hyperactivity disorder (ADHD) (OR 3·9, 95% CI 2·5–9·7). Clinical assessments of mental health diagnoses, such as depression (OR 4·7, 95% CI 3·6–6·1), anxiety (OR 4·1, 95% CI 3·2–5·3), and eating disorders (OR 2·8, 95% CI 2·1–3·6), were also found to be associated with having four or more ACEs, along with PTSD (OR 7·1, 95% CI 4·9–10·3) (Table 4).

**Table 4:** Association of physical and mental health diagnosis with the number of ACEs in Mexican adults aged 18 to 65 years.^a^

	1 ACE			2-3 ACEs			4 or more ACEs		
	OR	p	95% CI	OR	p	95% CI	OR	p	95% Cl
**Physical health self-report**									
Migraine	0·6	0·04	(0·4–0·9)	0·9	0·59	(0·6–1·2)	1·7	**0·002**	(1·2–2·5)
Diabetes mellitus	0·9	0·94	(0·6–1·4)	0·8	0·25	(0·5–1·1)	1·5	**0·04**	(1·0–2·2)
Obesity	0·6	0·05	(0·3–1·0)	0·8	0·43	(0·5–1·2)	1·8	**0·002**	(1·2–2·8)
Hypertension	0·8	0·45	(0·6–1·2)	0·8	0·20	(0·6–1·1)	1·6	**0·002**	(1·1–2·2)
Irritable bowel syndrome	0·7	0·29	(0·4–1·2)	1·0	0·93	(0·6–1·5)	1·8	**0·005**	(1·2–2·7)
Gastroesophageal reflux disease	0·6	0·12	(0·4–1·1)	1·2	0·23	(0·8–1·9)	1·6	**0·02**	(1·0–2·5)
Chronic pain	0·6	0·16	(0·3–1·1)	0·7	0·16	(0·4–1·1)	2·0	**0·004**	(1·2–3·2)
Insomnia	0·5	0·09	(0·2–1·1)	0·6	0·20	(0·3–1·2)	3·2	**<0·001**	(1·8–5·6)
**Mental health self-report**									
Depression	1·1	0·48	(0·7–1·9)	1·6	0·03	(1·0–2·6)	5·3	**<0·001**	(3·2–8·5)
PTSD	0·7	0·48	(0·4–1·5)	0·6	0·14	(0·3–1·1)	3·1	**<0·001**	(1·7–5·4)
Generalized anxiety disorder	0·5	0·19	(0·2–1·3)	1·1	0·76	(0·5–2·3)	3·3	**<0·001**	(1·5–6·9)
Suicidal ideation	0·1	0·001	(0·0–0·4)	0·4	0·007	(0·2–0·7)	8·6	**<0·001**	(4·9–14·8)
Non-suicidal self harm (Cutting)	0·4	0·12	(0·1–1·2)	0·5	0·14	(0·2–1·2)	5·5	**<0·001**	(2·5–11·9)
Alcoholism	0·4	0·02	(0·2–0·8)	1·0	0·83	(0·6–1·7)	2·8	**<0·001**	(1·7–4·8)
ADHD	0·7	0·53	(0·2–2·1)	0·5	0·27	(0·2–1·5)	3·9	**<0·001**	(2·5–9·7)
**Mental health clinically assessed**									
PTSD (PCL-5)	0·3	<0·001	(0·2–0·6)	3·2	<0·001	(0·9–9·6)	7·1	**<0·001**	(4·9–10·3)
Depression (PHQ-9)	0·5	0·01	(0·2–0·4)	1·0	0·07	(0·7–1·5)	4·7	**<0·001**	(3·6–6·1)
Generalized anxiety disorder (GAD-7)	0·9	0·09	(0·5–1·4)	1·4	0·08	(0·9–2·1)	4·1	**<0·001**	(3·2–5·3)
Eating disorders (SCOFF)	0·5	<0·001	(0·3–0·6)	0·7	0·07	(0·6–1·0)	2·8	**<0·001**	(2·1–3·6)

Statistical significance was determined with a bolded p-value <0·05. Reference group for comparison: 0 ACEs.

a Unadjusted logistic regression models.

We then adjusted the models based on sociodemographic factors such as age, sex, urbanity stratum, and wellness condition. Subsequently, another adjustment was performed using the BCEs score (Table 5). The model adjusted with sociodemographic factors revealed increased odds for physical diagnoses such as diabetes (OR 1·7, 95% CI 1·1–2·6), obesity (OR 1·9, 95% CI 1·2–2·9), hypertension (OR 2·0, 95% CI 1·4–2·8), and chronic pain (OR 2·1, 95% CI 1·3–3·4). Furthermore, the adjustment increased the odds of self-reported mental health diagnoses such as depression (OR 3·9, 95% CI 2·9–5·1), ADHD (OR 4·1, 95% CI 1·6–10·5), and alcoholism (OR 4·1, 95% CI 2·3–7·3), as well as clinically assessed diagnoses like PTSD (OR 7·3, 95% CI 5·0–10·6), anxiety (OR 4·2, 95% CI 3·2–5·5), and depression (OR 4·8, 95% CI 3·7–6·3). Being men reduced the odds of having migraine (OR 0·5, 95% CI 0·3–0·8), irritable bowel syndrome (OR 0·5, 95% CI 0·3–0·9), clinically assessed depression (OR 0·5 95% CI 0·4–0·8), and eating disorders (OR 0·4 95% CI 0·3–0·6) while increasing the odds of having self-reported diagnoses such as ADHD (OR 4·0 95% CI 1·5–10·4).

**Table 5:** Association of any physical and mental health diagnoses with experiencing four or more ACEs: Adjusted Models.^c^

	Raw model			Adjusted model^a^			Adjusted model^b^ (BCEs)		
	OR	p	95% Cl	OR	p	95% Cl	OR	p	95% Cl
**Physical health self-report**									
Migraine	1·7	0·002	(1·2–2·5)	1·7	0·002	(1·2–2·5)	**1·6**	0·01	(1·0–2·3)
Diabetes mellitus	1·5	0·04	(1·0–2·2)	1·7	0·01	(1·1–2·6)	1·7	0·008	(1·1–2·7)
Obesity	1·8	0·002	(1·2–2·8)	1·9	0·002	(1·2–2·9)	**1·8**	0·003	(1·2–2·8)
Hypertension	1·6	0·002	(1·1–2·2)	2·0	<0·001	(1·4–2·8)	**1·9**	0·001	(1·3–2·8)
Irritable bowel syndrome	1·8	0·005	(1·2–2·7)	1·8	0·005	(1·2–2·7)	1·5	0·06	(0·9–2·3)
Gastroesophageal reflux disease	1·6	0·02	(1·0–2·5)	1·6	0·02	(1·0–2·5)	1·4	0·09	(0·9–2·3)
Chronic pain	2·0	0·004	(1·2–3·2)	2·1	0·003	(1·3–3·4)	2·1	0·006	(1·3–3·5)
Insomnia	3·2	<0·001	(1·8–5·6)	3·2	<0·001	(1·8–5·7)	3·2	<0·001	(1·8–5·8)
**Mental health self-report**									
Depression	3·8	<0·001	(2·9–5·0)	3·9	<0·001	(2·9–5·1)	**3·5**	<0·001	(2·6–4·6)
Posttraumatic stress disorder	3·1	<0·001	(1·7–5·4)	3·0	<0·001	(1·7–5·3)	**2·9**	<0·001	(1·6–5·3)
Generalized anxiety disorder	3·6	<0·001	(2·4–5·5)	3·6	<0·001	(2·4–5·5)	**3·0**	<0·001	(1·9–4·7)
Suicidal ideation	8·6	<0·001	(4·9–14·8)	8·5	<0·001	(4·9–14·7)	**6·5**	<0·001	(3·6–11·6)
Non-suicidal self harm (Cutting)	5·5	<0·001	(2·5–11·9)	5·1	<0·001	(2·3–11·3)	2·5	0·03	(1·0–5·8)
Alcoholism	2·8	<0·001	(1·7–4·8)	4·1	<0·001	(2·3–7·3)	**3·7**	<0·001	(2·0–6·8)
Attention deficit and hyperactivity disorder	3·9	<0·001	(2·5–9·7)	4·1	0·003	(1·6–10·5)	**3·6**	0·01	(1·3–9·9)
**Mental health clinically assessed**									
PTSD (PCL-5)	7·1	<0·001	(4·9–10·3)	7·3	<0·001	(5·0–10·6)	**6·5**	<0·001	(4·4–9·6)
Depression (PHQ-9)	4·7	<0·001	(3·6–6·1)	4·8	<0·001	(3·7–6·3)	**4·2**	<0·001	(3·2–5·6)
Generalized anxiety disorder (GAD-7)	4·1	<0·001	(3·2–5·3)	4·2	<0·001	(3·2–5·5)	**3·8**	<0·001	(2·9–5·0)
Eating disorders (SCOFF)	2·8	<0·001	(2·1–3·6)	2·7	<0·001	(2·1–3·6)	**2·6**	<0·001	(0·9–1·0)

Bolded odds ratios (ORs) represent associations that decreased after adjusting for the BCE score.

a Age, gender, urban stratum, wellness condition.

b BCEs score.

c Statistical significance was determined with a p-value <0·01.

Upon making the following adjustment with the BCE score, we observed a slight reduction in the odds of migraines (OR 1·6, 95% CI 1·0–2·3), obesity (OR 1·8, 95% CI 1·2–2·8), and hypertension (OR 1·9, 95% CI 1·3–2·8). Additionally, there was a decrease in the odds related to almost every self-reported mental health diagnosis. This reduction in odds related to the BCE score was also observed in clinically assessed conditions like PTSD (OR 6·5, 95% CI 4·4–9·6), depression (OR 4·2, 95% CI 3·2–5·6), anxiety (OR 3·8, 95% CI 2·9–5·0), and eating disorders (OR 2·6, 95% CI 0·9–1·0).

## Discussion

This study represents the first attempt to determine the prevalence of ACEs and BCEs in a nationally representative sample of Mexican adults and to assess their impact on health outcomes. Using the ACE-IQ, we found that 88·2% of the adult Mexican population reported at least one ACE, while 22·6% reported four or more. This contrasts with United States data from the BRFSS 2011–2020, where 63·9% of U.S. adults reported at least one ACE, and 17·3% reported four or more.^1^ However, direct comparison is challenging due to differences in assessment tools. Our findings align with those reported by Ramirez Labbé et al. in Chile, where a prevalence of 88·9% for at least one ACE was found using the ACE-IQ, but also differ in their finding of 54·6% for four or more ACEs. These differences could be related to specific exposure to risks inherent to each country, and the similarities might be attributed to a shared social and cultural context in Latin America compared to other populations.^20^

The most frequent adversities encountered were physical neglect (58·6%), followed by emotional neglect (35·7%), absent or divorced parents (33·1%), and household violence (26·8%). These findings mirror those reported by Subramaniam et al. in Singapore, where emotional neglect (46·5%), parental absence (21·8%), and household violence (8·2%) were among the most frequently reported ACEs.^21^ The neglect categories found in Mexican adults are also the most frequent in Mexican adolescents. In a recent study by Casas-Muñoz, neglect was the most prevalent ACE, with 73·35% of adolescents reporting it.^22^

A history of childhood neglect has been associated with adverse outcomes in both non-clinical and clinical populations. Evidence from the Minnesota Longitudinal Study of Risk and Adaptation indicates that neglect is linked to higher rates of negative physical health outcomes in adulthood and increased cardiometabolic risk.^2^

Regarding abuse categories, sexual abuse reported by 14·5% was the most prevalent and was significantly more reported by women, followed by emotional abuse (9·2%) and physical abuse (7·2%). These findings are strikingly different from those in Mexican adolescents, as psychological abuse was the abuse category most frequently reported (37·31%), followed by physical abuse (30·72%) and sexual abuse (18·91%).^22^ These differences in the adolescent vs. adult population could be attributed to memory bias or even the normalization of emotional and physical abuse as a part of education and parenting. Abuse in all its forms has been extensively documented to have detrimental effects on both physical and mental health during childhood and adulthood, surpassing the impact of other adversities such as neglect. Specifically, sexual abuse has been linked to physical conditions like obesity, chronic pain, and infectious diseases such as HIV, as well as mental health diagnoses including depression, anxiety, PTSD, substance abuse, and suicidal attempts, among others.^2^

We found a 9·8% prevalence of community violence, 7·6% of bullying, and 2·4% of collective violence, with the latter being significantly more reported by men, similar to Chilean findings, and higher in urban areas compared to rural areas.^20^ This could be attributed to drug-related organized crime, a major contributor to urban violence in Mexico.^8^ Interestingly, Mexican adolescents reported 56·63% of community violence and 13·74% of collective violence.^22^ These differences could be attributed to the increasing rates of violent environments within the Mexican context.^8^ Collective violence linked to organized crime has previously been associated with poor mental health in children living near the Mexico–United States border.^23^ It may represent a context-specific risk factor for adversity among Mexican and Latin American children. The impact of the increasing rates of violence within the region warrant further longitudinal exploration.

On a positive side, the most frequently reported BCE was having at least one caregiver with whom participants felt safe (89·6%), consistent with findings from Narayan's study.^3^ Extensive research has linked supportive home environments to resilience. In this context, cohesive Latin American family models may enhance feelings of security and support, promoting positive functioning in adulthood.^9^

Feeling comfortable with oneself (87·8%) was the second most frequently reported BCE. This contrasts sharply with findings from the U.S., where lower frequencies were reported. A positive self-concept can serve as a protective factor, as it is associated with adaptive functioning in children who experience maltreatment.^3^

Enjoying school was the third most reported BCE, and was mostly reported by women. This suggests that school can function as a protective environment and serve as a crucial platform for identifying and delivering interventions to mitigate adversity and promote positive experiences.

Having four or more ACEs was associated with migraines, diabetes, obesity, hypertension, irritable bowel syndrome, gastroesophageal reflux disease, chronic pain, and insomnia. In Mexico, Flores-Torres described an association between having four or more ACEs and obesity, diabetes, and hypertension.^11^ Priego-Parra et al. have also described the association between ACEs and irritable bowel syndrome in a predominantly female Mexican population, where a higher prevalence of ACEs was found among people diagnosed with it.^24^

There is strong evidence highlighting the impact of adversity, especially during early childhood, a critical developmental period, on the activation of neurobiological pathways linked to physical and mental illness in adulthood. The stress response triggered by adversity is multi-faceted, involving various physiological systems, including endocrine, metabolic, immune, and cardiovascular systems. These systems interact, and the combined changes can represent a health burden, even if a single indicator does not predict health outcomes. Allostatic load captures the cumulative physiological burden of chronic stress exposure and can be associated with deleterious physical and mental health outcomes.^25^

It is worth mentioning that a high prevalence of depression (33·8%) was found in our study. This data relates to the last prevalence obtained in the National Health and Nutrition Survey in 2022, which reports 16·7% of depressive symptoms in young adults and 38·3% in older adults. Both data highly contrast with the reported prevalence of depression by the Mexican health secretary (5·6%) in 2022 based on public mental health services data. These differences can be attributed to mental health services accessibility problems within the country and warrant public health attention.^26^

There was an association between having four or more ACEs and conditions such as depression, anxiety, PTSD, non-suicidal self-harm, alcoholism, eating disorders, and ADHD. These associations are consistent with findings by Landa-Blanco et al. in the Honduran population, where higher scores of depression, anxiety, somatization, and alcohol use were related to higher ACE scores.^27^ Also, suicidal behavior related to the cumulative effect of ACEs and mental health problems has been described in Mexican adolescents, indicating an early onset and detrimental impact of ACEs on mental health.^28^

Adjusting for BCEs reduced the odds of having migraines, obesity, and hypertension. This reduction was also observed in the odds of having mental health diagnoses such as depression, anxiety, PTSD, suicidal ideation, non-suicidal self-harm, alcoholism, eating disorders, and ADHD. Huang et al. described that adults with 5–6 positive childhood experiences had a 34·6% lower risk of fair or poor self-related health, with higher positive childhood experiences associated with a lower psychiatric and physical diagnosis risk.^29^ BCEs have been found to counteract the effects of ACEs, reducing psychological distress, depressive symptoms, and suicidal ideation.^5^ These findings relate to mediation in core neurobiological mechanisms that can buffer stress and promote resilient functioning even after adversity.^4^

However, the protective influence of BCEs has shown mixed results. In South Africa, a study of young adults found that BCE scores were not associated with protection or reduction of the odds of having depression. Therefore, further analysis of their implications and temporal and social dynamics is needed.^30^

This study has strengths and limitations. One strength lies in its novelty as the first study to report the prevalence of ACEs and BCEs in adults within a nationally representative sample, encompassing randomized urban and rural areas. Consequently, the results can be extrapolated to the broader Mexican population, enabling cross-country comparisons within similar contexts. Additionally, by focusing on a population often underrepresented in ACE studies, this research contributes significantly to filling the knowledge gap regarding the Latin American population. Consequently, it aids in the formulation of screening methods, preventive strategies, intervention proposals, and the development of informed national and international public policies. These efforts align with global initiatives aimed at promoting equity and inclusive child welfare.

However, this study has limitations. First, its cross-sectional design constrains the ability to establish causality regarding the observed associations. Secondly, the retrospective recollection of ACEs in adulthood may induce recall bias. Also, data regarding belonging to specific indigenous groups was not collected, so disaggregation by ethnic groups is unavailable. Finally, women were more likely to participate in the study. Future research endeavors could address these limitations by collecting more male participants to ensure balance in sex and gender representation, collecting ethnic group specifications, replicating ACEs and BCEs screening, analyzing subgroups including sociodemographic variables, and exploring biomarkers associated with allostatic load and BCEs buffering mechanisms. These endeavors could provide insights into specific prevention and intervention targets, as well as help identify groups requiring tailored interventions.

In conclusion, adverse childhood experiences are prevalent in Mexico and exert a significant impact on physical and mental health outcomes. However, BCEs can mitigate their impact. A comprehensive approach that considers ACEs and BCEs in child welfare policies, both nationally and internationally, can contribute to the formulation of interventions aimed at preventing adversity and promoting positive experiences for children in middle-income countries.

## Contributors

DLR: Conceptualization, literature research, study design, data curation, data analysis, data interpretation, writing original draft, reviewing & editing, and figures. Accessed and verified all the data.

FCT: Conceptualization, literature research, study design, data curation, writing-review & editing. Accessed and verified all the data.

NYTS: Methodology, data curation, writing-review & editing.

IME: Data curation, writing-review & editing.

VMR: Study design, data collection supervision, methodology, writing-review & editing.

BMT: Study design, data collection supervision, methodology, writing-review & editing.

JRDI: Funding acquisition, conceptualization, literature research, study design, data curation, writing-review & editing. Accessed and verified all the data.

All authors had full access to all the data in the study and had final responsibility for the decision to submit for publication.

## Data sharing statement

Individual participant data after de-identification and study protocol will be available upon reasonable request and after approval of a proposal, with a signed data access agreement through the corresponding author, Julieta Rodriguez-de-Ita (julyrdz@tec.mx). It will be available immediately following publication with no end date.

## Declaration of interests

All the authors (DLR, FCT, NYTS, IME, VMR, BMT, and JRDI) declare no competing interests. DLR received support from Tecnologico de Monterrey Challenge-Based Research Funding Program 2022 to attend ISPCAN Congress 2023 and 2024 and give an oral presentation related to the study protocol.

## Appendix A Supplementary data

Translated abtract
