# Supplementary material for: Implications of adverse and benevolent childhood experiences on the physical and mental health of Mexican adults: a population-based study
Source: Lancet Reg Health Am. 2025 Apr 19;46:101092. doi: 10.1016/j.lana.2025.101092 (PMC12033956; doi:10.1016/j.lana.2025.101092)
Supplement: Translated abtract [file mmc1.docx]

Editor note: This translation in Spanish was submitted by the authors and we reproduce it as supplied. It has not been peer reviewed. Our editorial processes have only been applied to the original abstract in English, which should serve as reference for this manuscript.

**Resumen**

**Antecedentes:** Las experiencias adversas en la infancia (ACEs por sus siglas en inglés) impactan negativamente la salud física y mental. Existe información limitada sobre su influencia en América Latina y países de ingresos medios como México. Este estudio tuvo como objetivo determinar la prevalencia y el impacto de las ACEs y las experiencias benevolentes en la infancia (BCEs por sus siglas en inglés) en la salud de la población mexicana.

**Métodos:** De septiembre a noviembre de 2023, este estudio transversal reclutó una muestra representativa a nivel nacional de adultos de 18 a 65 años, seleccionados aleatoriamente de áreas urbanas y rurales. Se recopilaron datos sociodemográficos, ACEs, BCEs e historial de salud física y mental. También se realizaron evaluaciones clínicas de depresión, ansiedad, trastorno de estrés postraumático y trastornos de la conducta alimentaria.

**Resultados:** De 1448 participantes reclutados, 1115 (77%) eran mujeres, 1278 (88·2%) reportaron al menos una ACE, mientras que 328 (22·6%) reportaron cuatro o más. Negligencia física (840; 58·6%) y emocional (518; 35·7%) fueron las ACEs más frecuentes. Cuatro o más ACEs aumentaron las probabilidades de obesidad (OR 1·8, 95% IC 1·2-2·8), hipertensión (OR 1·6, 95%IC 1·1-2·2), depresión (OR 4·7, 95%IC 3·6-6·1) y ansiedad (OR 4·1, 95%IC 3·2-5·3), entre otras. Las BCEs más comunes fueron tener al menos un cuidador de apoyo (1298; 89·6%) y sentirse cómodo con uno mismo (1272; 87·8%). Las BCEs disminuyeron las probabilidades de padecer diagnósticos de salud física y mental.

**Interpretación**: Las ACEs tienen una alta prevalencia e impactan significativamente en la salud de la población mexicana. Las BCEs protegen contra estos efectos. Considerar las ACEs en las políticas públicas puede ayudar a establecer intervenciones para prevenir la adversidad y promover experiencias positivas en la niñez.

**Financiamiento:** Fundación FEMSA, Centro de Primera Infancia del Tecnológico de Monterrey y Fundación FEMSA y Tecnologico de Monterrey Challenge-Based Research Funding Program 2022.
