# Supplementary material for: Implications of adverse and benevolent childhood experiences on the physical and mental health of Mexican adults: a population-based study
Source: Lancet Reg Health Am. 2025 Apr 19;46:101092. doi: 10.1016/j.lana.2025.101092 (PMC12033956; doi:10.1016/j.lana.2025.101092)
Supplement: Supplementary Material [file mmc2.docx]

**Table S1 Prevalence of ACEs and BCEs in Mexican adults aged 18 to 65: Men vs Women comparison**

|  | **National Population ^a^** | | | **Men** | | | **Women** | | | **P ^b^** |
| --- | --- | --- | --- | --- | --- | --- | --- | --- | --- | --- |
| **ACEs** | N | % | 95% CI | N | % | 95% CI | N | % | 95% CI |  |
| Physical Neglect | 840 | 58·6 | (54-63·1) | 182 | 21·6 | (18·8-24·4) | 658 | 78·3 | (75·5-81·1) | 0·12 |
| Emotional Neglect | 518 | 35·7 | (31·8-39·7) | 123 | 23·7 | (20·0-27·4) | 395 | 76·2 | (72·5-79·9) | 0·66 |
| Parental Separation or Divorce | 480 | 33·1 | (30·3-38·7) | 113 | 23·5 | (19·6-27·2) | 367 | 76·5 | (72·7-80·3) | 0·82 |
| Household Member Treated Violently | 389 | 26·8 | (21·4-27·9) | 83 | 21·3 | (17·2-25·4) | 306 | 78·6 | (74·5-82·7) | 0·33 |
| Alcohol or Drug Abuser in the Household | 374 | 25·8 | (19·3-26·6) | 83 | 22·1 | (18·0-26·3) | 291 | 77·8 | (73·6-81·9) | 0·62 |
| Sexual abuse | 211 | 14·5 | (13·7-20·2) | 24 | 11·3 | (7·09-15·6) | 187 | 88·6 | (84·3-92·9) | **<0·001** |
| Community violence | 143 | 9·8 | (7·7- 11·2) | 33 | 23·0 | (16·1-29·9) | 110 | 76·9 | (70·01-83·8) | **0·001** |
| Emotional abuse | 134 | 9·2 | (6·9- 10·8) | 24 | 17·9 | (11·4-24·4) | 110 | 82·0 | (75·6-88·5) | 0·13 |
| Mental Disorders within the Household | 133 | 9·2 | (7·6- 11·3) | 32 | 24·0 | (16·8-31·3) | 101 | 75·9 | (68·6-83·2) | 0·78 |
| Bullying | 110 | 7·6 | (7·4- 11·9) | 32 | 29·0 | (20·4-37·2) | 78 | 70·9 | (62·7-79·5) | 0·13 |
| Physical Abuse | 105 | 7·2 | (4·9- 9·1) | 21 | 20·0 | (12·3-27·6) | 84 | 80·0 | (72·3-87·6) | 0·43 |
| Incarcerated Household Member | 85 | 5·8 | (4·3- 8·3) | 21 | 24·7 | (15·4-33·9) | 64 | 75·2 | (66·0-84·5) | 0·71 |
| Collective violence | 34 | 2·4 | (2·0- 4·7) | 19 | 55·8 | (39·1-72·5) | 15 | 44·1 | (27·4-60·8) | **<0·001** |
| **BCEs** |  |  |  |  |  |  |  |  |  |  |
| At least one caregiver whom you felt safe | 1298 | 89·6 | (88·1-92·6) | 301 | 23·1 | (20·3-24·8) | 997 | 76·8 | (72·7-77·4) | 0·83 |
| Liking or feeling comfortable with yourself | 1272 | 87·8 | (86·0- 90·3) | 303 | 23·8 | (21·4-26·1) | 969 | 76·1 | (73·8-78·5) | 0·08 |
| Enjoying school | 1244 | 86·0 | (83·5- 89·3) | 276 | 22·1 | (19·8-24·4) | 968 | 77·8 | (75·54-80·1) | **0·03** |
| Having good neighbors | 1221 | 84·3 | (81·0- 86·6) | 286 | 23·4 | (21·0-25·7) | 935 | 76·6 | (75·1-80·2) | 0·48 |
| Having predictable home routines | 1181 | 81·6 | (77·7- 85·1) | 268 | 22·6 | (20·2-25·0) | 913 | 77·3 | (74·96-79·7) | 0·41 |
| Having opportunities to have a good time | 1122 | 77·4 | (77·6- 83·0) | 288 | 25·6 | (23·1-28·2) | 834 | 74·3 | (71·7-76·9) | **<0·001** |
| Adult (not parent or caregiver) providing support | 1108 | 76·5 | (75·1- 81·4) | 261 | 23·5 | (21·0-26·0) | 847 | 76·4 | (73·99-78·9) | 0·47 |
| At least one good friend | 1083 | 74·8 | (73·1- 79·8) | 280 | 25·8 | (23·2-28·4) | 803 | 74·1 | (74·2-78·9) | **<0·001** |
| Beliefs that gave you comfort | 1061 | 73·3 | (70·1- 77·4) | 237 | 22·3 | (19·7-24·8) | 824 | 77·6 | (75·20-80·2) | 0·23 |
| At least one teacher who cared about you | 899 | 62·0 | (61·2- 68·3) | 219 | 24·3 | (21·5-27·1) | 680 | 75·6 | (74·00-78·9) | 0·15 |

^a^Total population= 1448 adults, representing 75,461 nationwide. ^b^ X^2^

# Protocol Summary

Prevalence of Adverse and Benevolent Childhood Experiences in the Mexican Population

**Introduction**

Early childhood experiences have gained recognition as an essential factor in later stages of life. Adverse childhood experiences, such as mistreatment and neglect or growing up with severe forms of family dysfunction, are a public health concern internationally due to their impact on the overall health of both children and adults. Various studies have investigated the molecular mechanisms through which early adversity influences the human body's physiology and alter biological and developmental processes. Early life adversity has far-reaching effects on neuroendocrine, immune, and metabolic physiology. These biological changes are believed to increase the risk of health problems in adults, both directly and indirectly. In Mexico, we have limited information regarding the prevalence and health impact of adverse childhood experiences and even less evidence of the mediating effect of positive childhood experiences and the extent to which they could serve as protective factors, both in the life experience and health impact. Existing studies have predominantly been conducted in populations with a different cultural context and health system characteristics, limiting their generalization. This makes it necessary to obtain inclusive information from samples that represent our population and that allow describing problems, needs, and associated factors. Obtaining an initial approach to the prevalence of adverse childhood experiences of adults in Mexico can provide fundamental information to establish a situational diagnosis that allows the subsequent creation and implementation of specific intervention strategies aimed at preventing the deleterious impact on the population's health. Additionally, having a solid knowledge base will allow the establishment of recommendations in clinical, social, and public policy settings in search of benefiting the integral well-being of the population. Therefore, the main objective of this protocol is to determine the prevalence of adverse and benevolent childhood experiences in a representative sample of adults in Mexico and to assess their association with physical and mental health.

**Methodology**

Type of study: Cross-sectional, descriptive, with national, urban, and rural locality representativeness.

The sampling was designed to represent the national population aged 18 to 65. The participants will provide information to understand the magnitude of exposure to adverse and positive experiences and their association with physical health and well-being. The fieldwork development stage is planned to be carried out from August to September 2023.

Study population**:** 1,200 men and women aged 18 to 65 years.

Training for Interviewers: Interviewers will be trained in applying instruments and psychological first aid.

Participants: The participants will be men and women aged 18 to 65 who meet the selection criteria (Inclusion criteria: participants are to be between 18 and 65 years old; exclusion criteria: intellectual disability, limited understanding of the data collection instruments).

Data collection instruments:

1. Sociodemographic characteristics of the household and the informant.
2. Adverse Childhood Experiences Questionnaire (ACE-IQ): Explores adverse childhood experiences such as intrafamily violence, early death of one of the parents, or bullying, through the International
3. Benevolent Childhood Experiences (BCE) scale: This scale explores positive experiences in the first 18 years of life, such as having at least one adult to trust, having good friends or neighbors, enjoying school, and having a stable home routine.
4. Late adversities throughout life include being expelled from school due to learning or behavior problems or committing a crime.
5. Questionnaire on prior physical health diagnosis: This 20-question section inquires about previous diagnoses made by a doctor, including diseases such as diabetes, cardiovascular diseases, hypertension, obstructive sleep apnea, and gastroesophageal reflux.
6. Questionnaire on prior mental health diagnosis: Inquires whether the interviewed person has been diagnosed by a doctor with any mental illness in their lifetime, such as depression, bipolar disorder, schizophrenia, post- traumatic stress disorder, anorexia, bulimia nervosa, etc.
7. Patient Health Questionnaire (PHQ-9): a 9-item screening questionnaire that assesses depressive symptoms in the last two weeks.
8. Generalized Anxiety Disorder 7-item questionnaire (GAD-7): A screening test to measure generalized anxiety symptoms in primary health care levels.
9. The PTSD Checklist for DSM-5 (PCL-5) Screens the presence of posttraumatic stress disorders.
10. SCOFF Questionnaire: Consists of five questions that evaluate the core characteristics of anorexia and bulimia nervosa.
11. Personal well-being and pro-social behavior: Inquiries about factors such as helping, sharing, caring, and empathizing.

Data analysis: Descriptive analysis of the variables of interest to report prevalences and proportions, a bivariate analysis on important study domains, and an analysis of significant differences between comparison subgroups. Analysis with multivariate statistical models is used to explore associations between adverse experiences and participants' health and well-being problems.

**Ethics and Informed Consent**

This study was approved by the Ethics Committee of the National Institute of Public Health (CI:1860). Adult participants will sign an informed consent form, which outlines the study's objectives and the type of information that will be collected. Participants will receive an informational brochure describing Adverse Childhood Experiences, how they affect people's well-being, and how to address them. This brochure will also include a list of phone numbers where they can get help in case of child abuse or gender violence. A helpline managed by a team of psychologists from the School of Medicine and Health Sciences of Tecnologico de Monterrey will be available 24/7 for interested people to receive free psychological care or physiological first aid.

# Study protocol full length

Prevalence of Adverse and Benevolent Childhood Experiences in the Mexican Population

## Introduction

Early childhood experiences are increasingly recognized as important factors that influence health later in life^1^. Adverse childhood experiences (ACEs)—such as abuse and neglect or severe family dysfunction—are a public health concern worldwide because of their effect on the overall health of both children and adults. In the United States, three in every five adults report at least one ACE, and four or more ACEs are associated with a two-fold risk of chronic respiratory diseases, heart disease, and cancer, as well as childhood difficulties such as poor school performance, behavioral changes or issues, deterioration of mental and physical health, recurrent infections, and obesity^2^. Multiple studies have suggested that ACEs have a cumulative effect and may affect health. This phenomenon is explained by the toxic stress response, which comprises neuroimmunoendocrine and circadian alterations that occur from early childhood^3^.

In 1998, the Centers for Disease Control and Prevention (CDC) and Kaiser Permanente conducted a study called “Relationship of Childhood Abuse and Household Dysfunction to Many of the Leading Causes of Death in Adults: The Adverse Childhood Experiences (ACE) Study,” where they documented the importance and health effects of ACEs. The study included >17,000 middle-class US citizens and evidenced that ACEs can affect

>60% of adults and contribute significantly to negative effects on their mental and physical health^4^.

The study found a strong dose–response relationship between exposure to ACEs and health implications in adulthood: adults who had lived in dysfunctional homes as children were at greater risk of developing diseases, such as heart attacks, cancer, chronic lung disease, fractures, and liver disease, as well as depression, anxiety, sleep disturbances, poor self-esteem, and poorer self-care strategies^5^. This cumulative effect may be attributed to ACEs catalyzing a series of biological adaptations that change the manner in which the brain, neuroendocrine response to stress, and immune system function both individually and collectively^6^.

*Biological Effects of Adverse Childhood Experiences*

ACEs can alter vital core neurobiological systems during vulnerable periods of development, leading to considerable changes in stress regulation and psychophysiological reactivity^7^. Because of their key role in regulating the dynamic stress response and perhaps because of a historical focus on these two systems, the hypothalamic–pituitary–adrenal axis and locus ceruleus/autonomic nervous system have been more extensively investigated and are considered more affected than other biological systems^8^.

Several studies have investigated the molecular mechanisms by which ACEs affect human physiology and alter biological and developmental processes^9^ and neuroendocrine, immune, and metabolic physiology^10^. These biological changes increase the risk of developing health conditions in adults, both directly and indirectly^11^. The concept of allostatic load is used to understand the processes that directly link ACEs and poor health

outcomes in adulthood^12^ ; according to allostatic load, when physiological systems—such as the nervous, endocrine, and immune systems—are chronically and repeatedly exposed to ACEs, they make repeated adjustments to maintain stability and, over time, may lose their ability to function properly, thereby generating an imbalance that negatively affects health.

Slopen et al. created an index comprising different biomarkers, including systolic and diastolic blood pressure, resting heart rate, and C-reactive protein (CRP) levels. Using this combination, they found that the association between cumulative biological risk and ACE changes according to the degree of exposure to the adversity and the individual’s social context^13^.

*Benevolent childhood experiences*

Although knowledge about ACEs and their effects on health has been increasing, little is known about exposure to positive childhood experiences and the extent to which they counteract risks and act as health protective factors. Research on developmental psychology has revealed that positive childhood experiences improve future social experiences and healthy relationships^14^. In addition, studies analyzing how childhood experiences affect adult roles have found that certain positive childhood experiences, such as interpersonal connectedness and school engagement, predicted significantly better productivity and responsibility in adulthood^15^. Similarly, familial warmth and extrafamilial support are related to more adaptive traits in adults, such as optimism^16^. Participants who scored higher than others on their index of positive childhood experiences had higher cardiovascular health scores and better self-care and health management behaviors than others^17^. Thus, these experiences may counteract the effects of ACEs and influence the development of adaptive behaviors and resilience that eventually improve an individual’s overall health.

*Measurement Strategies for Adverse Childhood Experiences*

The Adverse Childhood Experiences Scale (ACE Scale) developed in the first exploratory study is a brief and specific scale to measure adults’ exposure to ACEs. As a result, it provides a summary of the subtypes of experiences related to childhood trauma and mistreatment, including verbal/emotional, physical, and sexual abuse and emotional and physical neglect. Subsequent studies have added items to the assessment scales, including the search for adverse experiences, such as physical, verbal, and sexual abuse; household dysfunction; domestic violence; exposure to mental conditions; substance use; and criminal activity, neglect, and parental separation^18^. The addition of exposure to bullying and foster care has also been suggested^19^. Scales such as the Pediatric ACEs and Related Life Event Screener (PEARLS) have also been designed to assess both ACEs and related events, which may directly represent risk factors for toxic stress in children and adolescents^20^.

When studying ACEs, it is also important to document their associated health effects using standardized scales that serve as clinical screening with particular emphasis on general health (including obesity, cardiovascular disease, and psychosomatic and respiratory symptoms) and mental health outcomes (including depression, anxiety, post-traumatic stress disorder, and substance use in adults and neurodevelopment and behavior in children^21^).

Furthermore, measurement scales, such as the Benevolent Childhood Experiences (BCE) scale and the Child and Youth Resilience Measure (CYRM), are useful for documenting positive childhood experiences for adults and children. These instruments have good psychometric properties, have been translated into several languages, and are brief^22^.

*Prevention of Adverse Childhood Experiences*

To establish recommendations for addressing and preventing ACEs, the degree of ACE exposure (magnitude) and its health effects should be documented^23^. It is also crucial to understand how both adverse and positive childhood experiences interact in parents’ life experiences and how these experiences influence the lives of their children. Parents’ ACEs have been linked to compromised parenting skills, parental stress, and mental health disorders, such as depression and post-traumatic stress disorder that, in turn, pose a threat to children’s well- being^24^. In contrast, positive parental childhood experiences have been linked to better interpersonal resources, effective coping strategies, and resilience in the face of adversity^25^. This, in turn, may be a predictor of future adaptation in children and families, which merits further research to generate a basis for the creation of preventive strategies.

## Justification

In Mexico, information regarding the prevalence and health effects of ACEs is limited, and there is even less evidence of the mediating effect of benevolent childhood experiences and their role as protective factors in the life experience and health of adults and children.

Studies have been conducted predominantly in populations with different cultural contexts and health systems, thus limiting extrapolation. Therefore, it is necessary to obtain inclusive information from samples representing the Mexican population to describe their issues, needs, and associated factors.

A preliminary approach to understand the prevalence of ACEs Mexican population can provide crucial information for conducting a situational assessment that will help create and apply specific intervention strategies aimed at preventing negative implications on the population health. Similarly, having a solid knowledge base can help support recommendations in clinical, social, and public policy environments that benefit the population's overall well-being.

## Objectives

*General Objective*

To determine the prevalence of ACE and BCE exposure in a representative sample of adults in Mexico.

*Specific Objectives*

- To identify prior diagnoses of chronic diseases (diabetes, cardiovascular disease, and hypertension) or mental health conditions (depression, anxiety, and post-traumatic stress) and their association with ACEs and BCEs in adults aged 18–65 years.

*Research Question*

What is the prevalence of ACEs and BCEs and their association with previous diagnoses of chronic diseases and mental health conditions in Mexican adults (18-65 years old)?

## Methodology

*Type of study*

This is a cross-sectional, descriptive study with national representativeness, including urban and rural areas. The sampling was designed to represent 18–65-year-old adults in Mexico.

The 18–65-year age group provides information on exposure to adverse and positive experiences and their association with physical health and well-being.

The development stage of the fieldwork is scheduled to take place from August to September 2023.

*Study population*

The study population will comprise 1,200 men and women aged 18–65 years.

*Inclusion Criteria*

- Men and women aged 18–65 years.
- Participants who provided informed consent to participate in the study.

*Exclusion Criteria*

- intellectual disabilities that limit understanding of the data collection instruments.
- Refusal to continue interview.

*Sample size*

Sample size was calculated using the following formula:


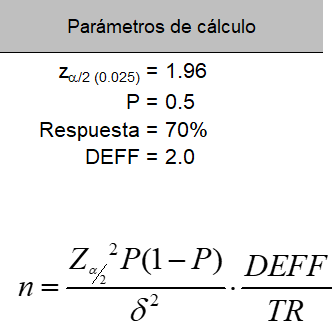


The sample size was estimated assuming maximum variance for the estimation of the prevalence of at least one early adversity (because there are no representative data for Mexico, the Survey of Adverse Childhood Experiences conducted in Chile, which reported 22% of cases of ACEs during childhood, was taken as a reference), with an estimation error of 5% and 95% confidence. A nonresponse rate of 30% and a design effect (DEFF) of 2 were considered. This resulted in a sample of 2,320 homes to be selected and visited.

*Sample selection procedure*

The selection of the population to be surveyed will be conducted in three stages. In the first stage, urban basic geographic units (AGEBs) according to the National Population and Housing Census 2020 will be used as primary sampling units (PSUs). Fifty-eight urban AGEBs (≥2,500 inhabitants) and 58 rural AGEBs (<2,500 inhabitants) will be randomly selected.

Probabilistic sampling will be applied proportionally to the number of adults aged 18–65 years within the selected PSUs.

In the second stage, four blocks within each AGEB or locality will be selected by simple random sampling. In the third stage, five households will be selected from each block using a systematic random selection procedure. Consequently, a sample of 20 households per AGEB will be considered.

Using this procedure, a total of 2,196 households will be sampled. Given that there are two domains (rural and urban areas), the expected sample size for an effective interview—considering the distribution of the population pyramid of the National Population and Housing Census 2020—is 689 people aged 18 to 65 years in urban areas and 709 in rural areas.

The following table presents the distribution of sampling units, the total number of households to be visited, and the expected final sample per group.

**Table 1. Distribution of samples for obtaining effective response**

| **Sampling units** | **Urban localities** | **Rural localities** | **Total** |
| --- | --- | --- | --- |

| Primary Sampling Units (PSUs) | 58 | 58 | 116 |
| --- | --- | --- | --- |
| Blocks selected per PSU | 4  Total 232 | 4  Total 232 | 464 |
| Households to be selected per block | 5  Total (1,160) | 5  Total (1,160) | 2,320 |
| Effective response of 18–65-year-old males and females only | 589 | 609 | **1,198** |

When a household has more than one 18–65-year-old adult, only one member meeting the selection criteria and wishing to participate in the study will be selected using a random number calculator included in the data entry form.

*Data Collection Instruments*

Data collection instruments are structured according to the population group to be interviewed. Questionnaire for 18–65-year-old adults

The respondents will be 18–65 years old and meet the selection criteria. This questionnaire includes the following sections (see Annex I):

*Section I:* It explores the sociodemographic characteristics of the household and respondent, such as marital status, educational level, occupation, monthly income, number of children, and ages of the children, and the public services available in the household and access to health services.

*Section II:* It enquires about the occurrence of a list of ACEs, such as domestic violence, early parent death, and bullying, using the Adverse Childhood Experiences International Questionnaire (ACE-IQ). The World Health Organization developed this questionnaire, which consists of 13 items that explore exposure to ACEs. It has a reliability of 0.854 Cronbach’s alpha and convergent validity of 0.85 when applied in community samples^26^.

*Section III* collects information on later-life adversities, such as expulsion from school for learning or behavioral problems and committing a crime.

*Section IV:* It examines previous diagnoses of mental health conditions, inquiring whether the interviewee was diagnosed by a physician with any mental illness such as depression, bipolar disorder, schizophrenia, post- traumatic stress disorder, anorexia, or bulimia nervosa.

*Section V:* It consists of the Patient Health Questionnaire (PHQ-9); created by Spitzer et al., this screening questionnaire consists of nine items assessing the presence of depressive symptoms (corresponding to DSM-IV criteria) in the past 2 weeks. Each item has a severity index from 0 to 3 on a Likert scale, where 0 is “never,” and 3 is “almost every day.” The usefulness of the PHQ-9 has been widely evaluated in both clinical and general

populations for detecting and diagnosing depressive symptoms, and it is a widely used tool in clinical practice. Its average filling time is 3.6 min. Its validity has been reported to be within satisfactory ranges, with a sensitivity of 88% and specificity of 88% with a cutoff point of 10 for major depression^27,28^. Its Mexican version has reliability coefficients of 0.77–0.89 and high internal consistency with a Cronbach’s alpha of 0.89^29^.

*Section VI:* It consists of the 7-item Generalized Anxiety Questionnaire (GAD-7), which was developed by Spitzer et al. as a screening test to measure symptoms of generalized anxiety at the primary healthcare level, and this test will be subsequently validated for use in the general population. It consists of a 7-point Likert-type scale with four response options (never, some days, half of the days, and almost every day), using which it determines the presence of anxiety symptoms in the last 2 weeks. A cutoff point of “10” provides adequate values of sensitivity (86.8%) and specificity (93.4%), with a Cronbach’s alpha of 0.86 reported in the Mexican population. Its average filling time is 2 min^30^.

*Section VII:* The Post-traumatic Stress Disorder Checklist for DSM-5 (PCL-5) was developed based on the diagnostic criteria proposed in the fifth version of the DSM, specifically criteria B, C, D, and E, which are evaluated using a Likert-type scale ranging from 0 (not at all) to 4 (extremely), in which the intensity of the symptoms described is identified. For its psychometric properties, several studies have focused on instrument reliability, analyses for convergent and discriminant validity, and factorial analyses. In these studies, the instrument showed high internal consistency (α = 0.94) and adequate test–retest reliability (r = 0.82), in addition to having adequate convergent (rs = 0.74 to 0.85) and discriminant (rs = 0.31 to 0.60) validity^31^.

*Section VIII:* This is a 20-question section that enquires about previous diagnoses of diseases, mainly chronic diseases such as diabetes, cardiovascular diseases, hypertension, obstructive sleep apnea, and gastroesophageal reflux.

*Section IX:* It includes the SCOFF Questionnaire, a five-item questionnaire that assesses the core features of anorexia nervosa and bulimia nervosa as a screening measure, to evaluate the presence of eating disorders. It has a combined sensitivity of 0.86 and specificity of 0.83^32^.

*Section X*: Using the BCEs questionnaire developed by Narayan A et al., it explores the occurrence of positive experiences in the first 18 years of life, such as having at least one adult to trust, having good friends or neighbors, enjoying school, and having a stable routine at home. This 10-item checklist dichotomously assesses exposure to positive experiences between the ages of 0 and 18, with a Cronbach’s alpha of 0.70^33^.

*Section XI:* Using the scale of prosociality in adults developed by Caprara et al., it enquires about personal well- being and prosocial behavior, analyzing factors such as helping, sharing, caring, and empathizing. This scale has 16 items scored on a Likert scale from 1 (never) to 5 (always), with a total score ranging from 16 to 80,

where higher scores indicate higher levels of prosocial behavior^34^. It has a reliability level of 0.90 and a Cronbach’s alpha of 0.78^3536^.

*Training of Fieldwork Personnel*

The training provided to fieldwork personnel will consist of three modules which will be delivered as follows:

1. Theoretical training
2. Hands-on training
3. Training on the application of interviewing techniques and data capture program
4. Training about the ethical considerations of the study

Experts will conduct training regarding the questionnaires' contents and relevant considerations to standardize the interviewers' knowledge on the application and on basic understanding of psychological first aid.

Specifically, the research group of the School of Medicine and Health Sciences of the Instituto Tecnológico de Estudios Superiores de Monterrey and the National Institute of Public Health (INSP) will provide the training. The fieldwork personnel will have an interviewer’s manual which has a detailed explanation of the procedures.

*Fieldwork Strategy*

Fieldwork will be conducted by a team from the National Surveys Directorate of the Evaluation and Survey Research Center (CIEE) of the INSP, which has extensive experience in conducting fieldwork at the national level.

The strategy for conducting the field work includes locating selected AGEBS and selecting homes where participants who meet the selection criteria can be located.

The interviewers will have tablets with which they can register information related to the questionnaires. All these procedures will be carried out in the homes of the participating population. If a participant does not have time, an appointment will be made.

The personnel in charge of data collection has a team of supervisors with extensive experience in group organization. These supervisors are key to the proper development of the fieldwork. Among their functions are

(a) preparation of work routes and logistics of fieldwork personnel (formation of teams, number of interviews to be conducted per day, activities to be performed by each member of the team, estimated time for transfers, etc.); (b) providing support to the interviewers if problems arise at the work sites; (c) quality control and backup of the information generated during the day; (d) periodic submission of the information to the technical support area for safekeeping and generation of progress reports; (e) preparation of weekly progress reports on fieldwork; and (f) collaboration with heads of the health and public safety secretariats of each state, municipality, and local authorities.

*Data Processing and Statistical Analysis*

Quality review and data cleaning will be performed immediately after data collection in the field, where the supervisors will verify that the interviews are obtained following the protocol reviewed during training. The data entry form is programmed to record only the response codes considered valid; if it detects codes considered invalid, the information is not recorded.

The data will be backed up daily by the supervisor of each team, who will send it to the technical support team twice a week. Subsequently, the technical support team will draft coverage reports to learn about the efficiency of the work and make the necessary adjustments to improve performance.

At the end of the fieldwork period, databases will be pooled, and incomplete interview records will be excluded to proceed with the information processing.

The statistical analysis of the information will consist of the following:

Descriptive statistics, such as frequencies and percentages, will be used to describe sociodemographic characteristics and the proportion of adults with more than one adverse and positive childhood experience.

Multiple models will be developed to determine the association between the number of ACEs and their association with chronic diseases or depressive symptoms in 18–65-year-old adults. Statistical significance will be determined for each model with a p value <0.05.

The descriptive statistics and statistical models will be adjusted according to the study design, PSU, selection stratum, and weights using the SVY module of STATA.

## Problems and Limitations

The main problem that the project may face is related to individuals’ participation. The following awareness-raising measures are also considered:

- *Interview dissemination:* The Ministry of Health will be asked to inform their state counterparts about the study and its dissemination within organizations to raise awareness among the population. In addition, dissemination through social networks will be conducted through the INSP website.
- *Consultation with authorities:* Before the start of activities in each locality, the authorities will be contacted—particularly those of health and public security organizations—to review the coverage of the operation and to provide an approximate time that will be spent in each locality.

Another limitation against the expected response rate is the level of public insecurity in some regions, which forces personnel to work in short shifts and conduct fieldwork operations more slowly. Nevertheless, we expect that the time allocated for the fieldwork will be sufficient to obtain the necessary information. In addition, care and prevention measures will be implemented to protect personnel’s integrity, such as

- *Identification of personnel:* The fieldwork team (coordinators, supervisors, and interviewers) will have an identity card with a photo, the INSP logo, and the contact number of the National Surveys Directorate. The personnel will be instructed to visibly wear their identity cards during the activities and provide the survey participants with the contact information of those responsible for the study when required. In addition, the personnel will wear clothing with the INSP logo. Furthermore, the

personnel are asked to carry other official identification documents, preferably the National Electoral Institute (INE) credential, and to show them if required by participants to increase their trust. The credentials that will identify the fieldwork personnel will be laminated and will have the registration number that identifies the project they are working on and the interviewer’s number.

- *Vehicle identification:* all vehicles used for the operation will bear the logo of the INSP and the Ministry of Health.
- *Fieldwork planning:* Before surveying each PSU, a desk review about the conditions of the area will be made, preferably by contacting those responsible for public safety, to determine the appropriate working hours, obtain public safety contact numbers, identify the appropriate size of the fieldwork teams for their protection, and in extreme cases, request the substitution of an area because it is considered to be at high security risk. The fieldwork personnel will be monitored by the supervisors, at a ratio of eight interviewers per supervisor. The supervisors must verify the location of the interviewers and their vehicle movements to and from the work areas. All interviewers and supervisors will carry cell phones to communicate with each other. Night activities will be avoided as much as possible. If the interviewer must make a night visit, they will be accompanied by the supervisor and transported in an official vehicle.
- *Risk identification:* A risk identification session will be included in the induction course, instructing the fieldwork team to avoid places where dangerous activities have been identified and explaining how to act when faced with challenging situations. In particular, they are instructed to avoid moving around during violent events, to seek shelter in the homes visited, not to remain in vehicles, to always carry their identity cards, and not to take out computer equipment in public areas. Before leaving a household, personnel should ensure that no suspicious vehicle is found (running and with several people inside, tinted windows, without license plates, etc.). When suspicious vehicles are found, the interviewers shall ask participants if they can stay in the house for an additional time and will contact their supervisors so that an official vehicle can pick them up. During interviews, interviewers should ensure that more than one person is present, particularly in the case of female personnel.
- *Transportation:* In addition to the preventive measures mentioned above, fieldwork teams will not be allowed to travel on roads of any type after 10:00 p.m.

## Funding

This research project will be carried out with the financial support of Fundación FEMSA and Instituto Tecnológico y de Estudios Superiores de Monterrey.

## Work Schedule

Activities are scheduled as follows:

**Work schedule for the project: “Prevalence of Adverse Childhood Experiences and their Association with Biological Markers of Toxic Stress in Parents and 3–5-year-old Children in Mexico.”**

| **Activity** | **2023** | | | | | | | | | **2024** | | |
| --- | --- | --- | --- | --- | --- | --- | --- | --- | --- | --- | --- | --- |
|  | **April** | **May** | **June** | **July** | **August** | **September** | **October** | **November** | **December** | **January** | **February** | **March** |
| Review of the protocol by the  Ethics, Research, and Biosafety Committees of the INSP |  |  | X |  |  |  |  |  |  |  |  |  |
| Personnel recruitment and  selection |  |  |  | X |  |  |  |  |  |  |  |  |
| Training of fieldwork personnel |  |  |  |  | X |  |  |  |  |  |  |  |
| Update to the Secretaries of Health and Public Safety on the project  schedule |  |  |  |  | X |  |  |  |  |  |  |  |
| Conducting fieldwork |  |  |  |  | X | X |  |  |  |  |  |  |
| End of fieldwork |  |  |  |  |  | X |  |  |  |  |  |  |
| Database compilation and cleaning |  |  |  |  |  | X |  |  |  |  |  |  |
| Data processing and preparation of  the first working report |  |  |  |  |  | X |  |  |  |  |  |  |
| Preparation of documents on  project results for decision-makers |  |  |  |  |  |  | X |  |  |  |  |  |
| Presentation of results to the  scientific community and civil society organizations |  |  |  |  |  |  |  | X |  |  |  |  |
| Preparation and submission of the first scientific paper based on  project results |  |  |  |  |  |  |  |  | X | X | X | X |

14

## Ethical Considerations

Study participants of legal age must sign an informed consent letter that states the objectives of the study and the type of information that will be collected from each group of participants. Consent letters will comply with the guidelines established by the Research Ethics Committee of the INSP. The interviewers will explain the study to potential participants, including its objective, voluntary nature, interview duration, procedures, risks, and benefits (Annex IV).

There are no inherent direct risks associated with participation in the research beyond those previously established in the General Health Act on Health Research, Article 17. This Act states that research with minimal risk includes “prospective studies employing data collection through common procedures in physical or psychological examinations of routine diagnosis or treatment, which include psychological tests to individuals or groups in which the participant's behavior is not manipulated.”^37^

Study participants will receive a brochure describing ACEs, how they affect people’s well-being, and how to deal with them. They will also find a list of telephone numbers where they can obtain help in case of abuse or gender violence and a telephone helpline run by a team of psychologists from the School of Medicine and Health Sciences of the Instituto Tecnológico y de Estudios Superiores de Monterrey, where free psychological care and crisis intervention will be provided (see Annex V).

*Confidentiality and Data Use*

Safeguarding of participants’ information will be carried out in accordance with the General Act for the Protection of Personal Data in Possession of Obligated Subjects. Only the interviewers will have access to the electronic devices in which the information will be captured through a specific folio. During the analysis, the identities of the respondents will be concealed. All databases will be safeguarded by the researchers responsible for the project.

## References

- 1. Petruccelli, K., Davis, J., & Berman, T. (2019). Adverse childhood experiences and associated health outcomes: A systematic review and meta-analysis. *Child abuse & neglect*, *97*, 104127. https://doi.org/10.1016/j.chiabu.2019.104127
  2. Merrick, M. T., Ford, D. C., Ports, K. A., Guinn, A. S., Chen, J., Klevens, J., et al. (2019). Vital signs: Estimated proportion of adult health problems attributable to adverse childhood experiences and implications for prevention—25 States, 2015–2017. MMWR. Morbidity and Mortality Weekly Report, 68, 999–1005
  3. Hughes, K., Bellis, M. A., Hardcastle, K. A., Sethi, D., Butchart, A., Mikton, C., et al. (2017). The effect of multiple adverse childhood experiences on health: A systematic review and meta-analysis. The Lancet Public Health, 2(8), e356–e366.
  4. Berens, A. E., Jensen, S. K. G., & Nelson, C. A. (2017). Biological embedding of childhood adversity: From physiological mechanisms to clinical implications. BMC Medicine, 15(1). https://doi.org/10.1186/s12916-017-0895-4. BioMed Central Ltd.
  5. Oh, D. L., Jerman, P., Silv ́erio Marques, S., Koita, K., Purewal Boparai, S. K., Burke Harris, N., et al. (2018). Systematic review of pediatric health outcomes associated with childhood adversity. BMC Pediatrics, 18(1), 83
  6. Thakur, N., Hessler, D., Koita, K., Ye, M., Benson, M., Gilgoff, R., Bucci, M., Long, D., & Burke Harris, N. (2020). Pediatrics adverse childhood experiences and related life events screener (PEARLS) and health in a safety-net practice. Child abuse & neglect, 108, 104685.
  7. Agorastos, A., Pervanidou, P., Chrousos, G. P., & Baker, D. G. (2019). Developmental Trajectories of Early Life Stress and Trauma: A Narrative Review on Neurobiological Aspects Beyond Stress System Dysregulation. *Frontiers in psychiatry*, *10*, 118.
  8. Danese A, J Lewis S. Psychoneuroimmunology of early-life stress: the hidden wounds of childhood trauma? *Neuropsychopharmacology*. (2017) 42:99–114.
  9. Hertzman, C. 2012. Putting the concept of biological embedding in historical perspective. Proc. Natl. Acad. Sci. USA. 109 (2), 17160–17167.
  10. Zelinski EL, Deibel SH, McDonald RJ. The trouble with circadian clock dysfunction: Multiple deleterious effects on the brain and body. *Neurosci Biobehav Rev.* (2014)
  11. Berens, A.E., Jensen, S.K.G., Nelson, C.A. 2017. Biological embedding of childhood adversity: from physiological mechanisms to clinical implications. BMC. Med. 15 (1), 135.
  12. Korotana, L.M., Dobson, K.S., Pusch, D., Josephson, T., 2016. A review of primary care interventions to improve health outcomes in adult survivors of adverse childhood experiences. Clin. Psychol. Rev.
  13. Hantsoo, L., Jašarević, E., Criniti, S., McGeehan, B., Tanes, C., Sammel, M. D., Elovitz, M. A., Compher, C., Wu, G., & Epperson, C. N. (2019). Childhood adversity impact on gut microbiota and inflammatory response to stress during pregnancy. *Brain, behavior, and immunity*, *75*, 240–250. https://doi.org/10.1016/j.bbi.2018.11.005
  14. Beach, S.R., Brody, G.H., Lei, M.K., Gibbons, F.X., Gerrard, M., Simons, R.L., et al. 2013. Impact of child sex abuse on adult psychopathology: a genetically and epigenetically informed investigation. J. Fam. Psychol.
  15. Friedman, E.M., Karlamangla, A.S., Gruenewald, T.L., Koretz, B., Seeman, T.E., 2015. Early life adversity and adult biological risk profiles. Psychosom Med. 77 (2), 176-185
  16. Narayan, A. J., Rivera, L. M., Bernstein, R. E., Harris, W. W., & Lieberman, A. F. (2018). Positive childhood experiences predict less psychopathology and stress in pregnant women with childhood adversity: A pilot study of the benevolent childhood experiences (BCEs) scale. Child Abuse & Neglect, 78, 19–30.
  17. Kosterman, R., Mason, W. A., Haggerty, K. P., Hawkins, J. D., Spoth, R., & Redmond, C. (2011). Positive childhood experiences and positive adult functioning: Prosocial continuity and the role of adolescent substance use. Journal of Adolescent Health, 49(2), 180–186.
  18. Slopen, N., Chen, Y., Guida, J. L., Albert, M. A., & Williams, D. R. (2017). Positive childhood experiences and ideal cardiovascular health in midlife: Associations and mediators. Preventive Medicine, 97, 72–79.
  19. Stone LL, Otten R, Engels RCME, Vermulst AA, Janssens JMAM. Psychometric properties of the parent and teacher versions of the strengths and difficulties questionnaire for 4- to 12-Year-olds: A review. Vol. 13, Clinical Child and Family Psychology Review. 2010. p. 254–74.
  20. Neeta Thakur, Danielle Hessler, Kadiatou Koita, Morgan Ye, Mindy Benson, Rachel Gilgoff, Monica Bucci, Dayna Long, Nadine Burke Harris, Pediatrics adverse childhood experiences and related life events screener (PEARLS) and health in a safety-net practice, Child Abuse & Neglect, Volume 108,2020,104685,ISSN 0145-2134,https://doi.org/10.1016/j.chiabu.2020.104685.
  21. Peter F. Cronholm, Christine M. Forke, Roy Wade, Megan H. Bair-Merritt, Martha Davis, Mary Harkins-Schwarz, Lee M. Pachter, Joel A. Fein, Adverse Childhood Experiences: Expanding the Concept of Adversity, American Journal of Preventive Medicine, Volume 49, Issue 3, 2015, Pages 354- 361,ISSN 0749-3797, https://doi.org/10.1016/j.amepre.2015.02.001
  22. Ungar, M., & Liebenberg, L. (2011). Assessing resilience across cultures using mixed methods: Construction of the child and youth resilience measure. Journal of Mixed Methods Research, 5, 126–

149. https://doi.org/10.1177/1558689811400607.

- 1. Merrick, J. S., & Narayan, A. J. (2020). Assessment and screening of positive childhood experiences along with childhood adversity in research, practice, and policy. Journal of Children and Poverty. https://doi.org/10.1080/10796126.2020.1799338.
  2. Narayan, A. J., Lieberman, A. F., & Masten, A. S. (2021). Intergenerational transmission and prevention of adverse childhood experiences (ACEs). *Clinical psychology review*, *85*, 101997. https://doi.org/10.1016/j.cpr.2021.101997
  3. Narayan, A. J., Rivera, L. M., Bernstein, R. E., Harris, W. W., & Lieberman, A. F. (2018). Positive childhood experiences predict less psychopathology and stress in pregnant women with childhood adversity: A pilot study of the benevolent childhood experiences (BCEs) scale. Child Abuse and Neglect, 78, 19–30. https://doi.org/ 10.1016/j.chiabu.2017.09.022.
  4. Pace, C. S., Muzi, S., Rogier, G., Meinero, L. L., & Marcenaro, S. (2022). The Adverse Childhood Experiences - International Questionnaire (ACE-IQ) in community samples around the world: A systematic review (part I). Child abuse & neglect, 129, 105640.
  5. Spitzer RL, Kroenke K, Williams JB. Validation and utility of a self-report version of PRIME- MD: the PHQ primary care study. Primary Care Evaluation of Mental Disorders. Patient Health Questionnaire. JAMA [Internet]. 1999 Nov 10 [cited 2019 Dec 10];282(18):1737– 44. Available from: <http://www.ncbi.nlm.nih.gov/pubmed/10568646>
  6. Martin A, Rief W, Klaiberg A, Braehler E. Validity of the Brief Patient Health Questionnaire Mood Scale (PHQ-9) in the general population. Gen Hosp Psychiatry. 2006 Jan;28(1):71–7. 21.
  7. Familiar, I., Ortiz-Panozo, E., Hall, B., Vieitez, I., Romieu, I., Lopez-Ridaura, R., & Lajous, M. (2015). Factor structure of the Spanish version of the Patient Health Questionnaire-9 in Mexican women. International journal of methods in psychiatric research, 24(1), 74–82. https://doi.org/10.1002/mpr.1461
  8. Spitzer RL, Kroenke K, Williams JBW, Löwe B. A brief measure for assessing generalized anxiety disorder: The GAD-7. Arch Intern Med. 2006 May 22;166(10):1092–7.
  9. Durón-Figueroa R, Cárdenas-López G, Castro-Calvo J, Rosa-Gómez AD la. Adaptación de la Lista Checable de Trastorno por Estrés Postraumático para DSM-5 en Población Mexicana. Acta Investig psicológica. 2019;9(1):26–36.
  10. Pace, C. S., Muzi, S., Rogier, G., Meinero, L. L., & Marcenaro, S. (2022). The Adverse Childhood Experiences - International Questionnaire (ACE-IQ) in community samples around the world: A systematic review (part I). Child abuse & neglect, 129, 105640.
  11. Narayan, A. J., Rivera, L. M., Bernstein, R. E., Harris, W. W., & Lieberman, A. F. (2018). Positive childhood experiences predict less psychopathology and stress in pregnant women with childhood adversity: A pilot study of the benevolent childhood experiences (BCEs) scale. Child abuse & neglect, 78, 19–30.
  12. Caprara, G. V., Steca, P., Zelli, A., & Capanna, C. A new scale for measuring adults’ prosocialness. European Journal of Psychological Assessment, 2005. 21(2), 77-89.
  13. Martí-Vilar, M., Merino-Soto, C., & Rodriguez, L. M. (2020). Measurement Invariance of the Prosocial Behavior Scale in Three Hispanic Countries Frontiers in psychology, 11, 29. https://doi.org/10.3389/fpsyg.2020.00029
  14. Luengo Kanacri BP, Eisenberg N, Tramontano C, Zuffiano A, Caprara MG, Regner E, Zhu L, Pastorelli C and Caprara GV (2021) Measuring Prosocial Behaviors: Psychometric Properties and Cross- National Validation of the Prosociality Scale in Five Countries. Front. Psychol. 12:693174. doi: 10.3389/fpsyg.2021.693174
